# Multidisciplinary Therapy to Target Obesity and Its Complications in Adult Population: A Narrative Review

**DOI:** 10.1111/obr.70093

**Published:** 2026-01-15

**Authors:** Ana Raimunda Dâmaso, Flávia Campos Corgosinho, Deborah Cristina Landi Masquio, Nayra Figueiredo, Fabiana Kattah, Cintia Cercato, Lian Tock, Raquel Munhoz da Silveira Campos

**Affiliations:** ^1^ Post‐Graduate Program of Nutrition Federal University of São Paulo ‐ Paulista Medicine School ‐ UNIFESP‐EPM São Paulo Brazil; ^2^ Brazilian Association of Study on Obesity and Metabolic Syndrome ABESO São Paulo Brazil; ^3^ Brazilian National Council for Scientific and Technological Development (CNPq) Brasília Brazil; ^4^ Post‐Graduate Program in Nutrition and Health ‐ Nutrition Faculty Federal University of Goiás ‐ UFG Goiânia Brazil; ^5^ Post‐Graduate Program in Science and Health ‐ Medicine Faculty Federal University of Goiás ‐ UFG Goiânia Brazil; ^6^ Post‐Graduate Program of Professional Nutrition: From Birth to Adolescence Centro Universitário São Camilo São Paulo Brazil; ^7^ Post‐Graduate Program of Endocrinology University of São Paulo ‐ Medicine School ‐ FMUSP São Paulo Brazil; ^8^ Post‐Graduate Program of Interdisciplinary in Health Sciences Federal University of São Paulo ‐ Campus Baixada Santista – UNIFESP‐CBS São Paulo Brazil

**Keywords:** adipose tissue metabolism, behavior counseling, energy balance, inflammation, metabolic syndrome, multidisciplinary therapy, nonalcoholic fatty liver diseases

## Abstract

Obesity as a chronic and multifactorial disease requires a multidisciplinary team acting together in a holistic multitarget intervention. Multidisciplinary therapy targeting obesity and its complications includes physical exercise, nutritional, and behavior counseling. When lifestyle changes are not sufficient in adults, medication prescription and bariatric surgery may be recommended. The aim of this narrative review is to summarize the underlying mechanisms and effects of multidisciplinary therapy in the control of obesity and its complications in adults. [Correction added on 21 January 2026, after first online publication: The previous sentence has been corrected in this version.] All types of clinical studies developed in an adult population were included. Different types of multidisciplinary therapy (short‐ and long‐term) in the primary and secondary settings were identified. Multidisciplinary therapy seems to be useful in the control of adverse effects of obesity, including reductions in biomarkers of inflammation, cardiometabolic risk factors, dyslipidemia, diabetes, hypertension, metabolic syndrome, nonalcoholic fatty liver diseases, atherosclerosis, cardiovascular disease, obstructive sleep apnea, and psychosocial outcomes. These results highlight the importance of targeting obesity and its complications in a holistic approach with a multidisciplinary team acting together.

## 1 Introduction

The World Health Organization (WHO) defines obesity as a multifactorial disease associated with a complication affecting quality of life and reducing life expectancy [1]. The global prevalence of obesity in adults is increasing around the world. Recent studies have shown that both high‐income and low‐ to middle‐income countries are experiencing rises in obesity rates [2, 3, 4].

Data from 3663 population‐based studies, involving 222 million individuals across 200 countries, revealed that the prevalence of obesity in adults rose between 1990 and 2022 in 188 countries (94%) for women and in all but one country for men. Additionally, the number of women and men with obesity in 2022 was 504 million and 374 million, respectively, which represent an increase of 377 million and 307 million, respectively, from 1990. The obesity rates increased by more than 20 percentage points in 49 countries (25%) among women and in 24 countries (12%) among men [4]. In the United States, 172 million adults (aged ≥ 25 years) are overweight or have a diagnosis of obesity. In 2050, projections indicate that the total number of adults with obesity is expected to reach 213 million, presenting a prevalence of 58.8% in women and 55.3% in men [2].

The main components behind the rising prevalence of obesity include behavioral factors, exercise and dietary patterns, and multifactorial causes affecting all of society. Factors commonly associated with sedentarism and food choices included age, gender, marital status, working hours, food security, and socioeconomic status [5, 6, 7, 8, 9, 10, 11, 12, 13, 14, 15, 16, 17, 18, 19].

Complications associated with obesity, especially severe and central obesity, may result from a subclinical inflammatory process leading to increased cardiometabolic risk, dyslipidemia, diabetes, hypertension, metabolic syndrome, nonalcoholic fatty liver disease, atherosclerosis, cardiovascular disease (CD), some types of cancer, polycystic ovary syndrome, psychosocial stress, mood disorders, obstructive sleep apnea, and others [17, 20, 21, 22, 23, 24, 25]. Moreover, people with severe obesity and hypothalamic inflammation may have a disruption in the control of inflammatory processes and the neuroendocrine regulation of energy balance [26, 27, 28].

Management of obesity and its complications may require the use of antiobesity medications, sometimes in long term during life, that is, for the control of diabetes and CDs [8, 29, 30, 31]. Antiobesity medications can improve adherence to a low‐calorie diet by decreasing appetite, increasing satiation, and modulating hedonic regulation of food intake [32]. Different guidelines recommend medication use for the adult population with BMI above 30 kg/m^2^ or BMI above 27 kg/m^2^ with at least one altered metabolic and functional condition or complication of obesity. Some Asia‐Pacific guidelines propose lower BMI thresholds, reflecting that Asian people develop obesity‐related complications at lower BMI than non‐Asian people [33]. For example, the 2009 consensus statement recommends pharmacotherapy in Asian Indian adults with BMI ≥ 27 kg/m^2^ or ≥ 25 kg/m^2^ in the presence of complications [34], while the South Korean guideline published in 2020 suggests a BMI threshold of 25 kg/m^2^ [2, 35, 36, 37, 38, 39].

The Food and Drug Administration (FDA) has approved several antiobesity medications with distinct mechanisms of action. These include Orlistat (a pancreatic and gastric lipase inhibitor that reduces dietary fat absorption), Bupropion/Naltrexone (a combination of a dopamine and norepinephrine reuptake inhibitor with an opioid receptor antagonist that decreases appetite and modulates reward pathways), Liraglutide and Semaglutide (GLP‐1 receptor agonists that enhance satiety through stimulation of pro‐opiomelanocortin [POMC] neurons, delay gastric emptying, and reduce appetite), Tirzepatide (a dual GIP and GLP‐1 receptor agonist that potentiates incretin effects and promotes weight loss through appetite suppression and enhanced insulin sensitivity), Setmelanotide (a MC4 receptor agonist effective in rare genetic forms of obesity), and Phentermine/Topiramate (a combination of a sympathomimetic amine and a GABA receptor modulator that suppresses appetite and increases energy expenditure). There are medications in phase 1 and 2 trials, including fibroblast growth factor (FGF) analog, long‐acting amylin analog, oxytocin receptor agonist, plasma peptide YY (PYY) 3‐36 analog, and mast cell stabilizer [39, 40, 41, 42, 43, 44, 45, 46, 47, 48, 49, 50, 51, 52].

Bariatric surgery is considered for severe obesity of BMI > 35 kg/m^2^ not responding to medications *and* multidisciplinary lifestyle approach. Bariatric surgery promotes a decrease in body weight, body fat mass, cardiometabolic risk factors, and subclinical myocardial injury and to increase activation of the cardiac natriuretic peptide system. These improvements seem mediated by a reduction in the leptin/adiponectin ratio, favoring a decrease in the proinflammatory state related to obesity [53, 54, 55, 56, 57].

Undesirable weight regain may occur after pharmacotherapy or bariatric surgery, particularly if people do not improve their lifestyle. Therefore, a multidisciplinary approach still plays an important role even in the long term [58, 59, 60, 61]. Each new attempt to lose weight, sometimes referred to as the yo‐yo effect, tends to be more challenging due to inherent difficulties in regulating the neuroendocrine mechanisms of whole‐body homeostasis [62, 63]. Unfortunately, the yo‐yo effect is a commonly observed response, not only after bariatric surgery and pharmacotherapy but also after lifestyle interventions like exercise training, caloric restriction, and behavior counseling.

Obesity is a disease of difficult control that requires a long‐term, structured, multidisciplinary therapy approach, including physiological and counseling considerations to foster lifestyle changes in exercise, quality of diet, and behavior [64, 65]. The aim of the present narrative review is to provide new evidence on the underlying mechanisms and effects of multidisciplinary therapy in obesity and its complications among adults.

## 2 Methods

For this narrative review, all types of clinical studies (randomized controlled trial studies, nonrandomized controlled trial studies, prospective studies, observational studies, and longitudinal studies) developed in an adult population were eligible. We explored the main objectives and mechanisms of action proposed in each intervention and covered outcomes resulting from multidisciplinary therapy targeting adults with obesity.

### 2.1 Data and Search Strategy

We searched Medline up to May of 2025, including the two structured search strings in Boxes 1 and 2. We consulted experts for relevant references. We also searched references of eligible studies and other studies through “snowballing.”

Box 1 **Search strategy based on interdisciplinary interventions definitions.**

**Table:** 

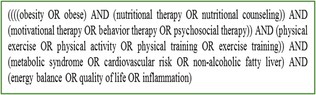

Box 2 **Search strategy based on isolated interventions definitions.**

**Table:** 

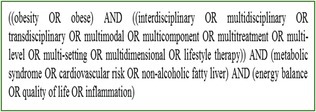

### 2.2 Eligibility Criteria

#### 2.2.1 Participants

Adults in all their diversity aged above 18 years old with obesity (as defined by trialists).

#### 2.2.2 Multidisciplinary Therapy

**FIGURE 1:**
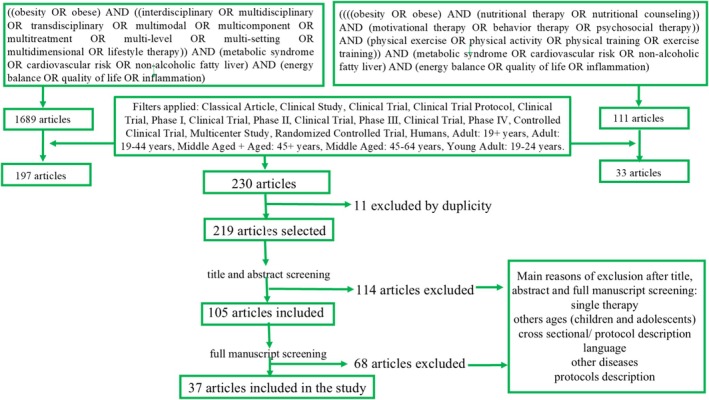
Flowchart.

Multidisciplinary therapy, in the context of obesity and its complications, can be considered as a team with two or more health workers from different health backgrounds working together with the objective to promote a holistic approach; covering a minimum of two components of lifestyle behavior, that is, physical activity/exercise, dietary habits, and/or psychological/behavior changing approach, in the same protocol associated with clinical intervention [66]. Studies could be in the community, family, clinical hospital, or multicenter setting. We excluded single therapy (only nutrition or only physical exercise intervention or supplementation therapies), cross‐sectional/protocol description studies, and studies including children and adolescents (Figure 1) [66]. Studies could be in the community, family, clinical hospital, or multicenter setting. We excluded single therapy (only nutrition or only physical exercise intervention or supplementation therapies), cross‐sectional/protocol description studies, and studies including children and adolescents (Figure 1).

#### 2.2.3 Outcomes

The outcomes assessed include body mass, body composition, adipose tissue, neuroendocrine regulation of energy balance, quality of life, biomarkers of inflammation, diabetes, nonalcoholic fatty liver diseases (NAFLD), cardiovascular risk, and metabolic syndrome.

#### 2.2.4 Selection Process

Titles and abstracts and full‐text screening were completed in duplicate. Discrepancies were solved by consensus or consulting a third author.

### 2.3 Data Extraction

We extracted information on authors, countries, study design, sample size, age, variables, multidisciplinary therapy, type of approach, sessions (frequency and duration), length (short‐ and long‐term, from 1 to 3 months and from 4 months to 2 years or more, respectively), and follow‐up.

### 2.4 Data Synthesis

According to the select studies, it was made a narrative summary of the findings considering the effects of multidisciplinary therapy in the control of obesity and its complications in adults.

## 3 Results

### 3.1 Overview of Studies

Summary of the results can be found in Figure 1 and Tables S1–S3. The Medline search resulted in 1800 potentially eligible references; 230 records were retained (Figure 1). After removal of 11 duplicates, we screened 219 references for title and abstract; of these, 114 were excluded (single therapy: only nutrition, only supplementation, only exercise intervention; children and adolescents; cross‐sectional/protocol description; other diseases). Of the remaining 105 studies, 68 were excluded after full‐text assessment.

A total of 37 articles remained (Figure 1). Of these, 29.7% (*n* = 11) originated from the Americas, 56.8% (*n* = 21) from Europe, 5.4% (*n* = 2) from Asia, and 8.1% (*n* = 3) from Oceania.

### 3.2 Study Characteristics and Interventions

Among the 37 articles selected in the present review, the different study designs included 25 randomized controlled trial studies, 5 nonrandomized controlled trial studies, 2 prospective studies, 3 observational studies, and 2 longitudinal studies. Sample size ranged from 16 individuals (women = 11, men = 5) to 8296 (women = 6111, men = 2185). Age ranged from 18 to 85.

Regarding interventions, 11 studies were based on group and individual intervention protocols; 18 studies were only group interventions; and eight were only individual interventions (Tables S2A and S2B). Duration ranged from 3 weeks to 2 years. The most common frequency of the sessions was two to three times per week with a minimal duration of approximately 20 min, although some studies reported a minor or major frequency/session duration. Ten studies had follow‐ups, with a minimum of 4 months and a maximum of 4 years. In addition, dropout rates were reported in 32 studies with a variation from 0% to 65.3% (Tables S2A and S2B).

### 3.3 Outcomes

The impact of multidisciplinary therapy is summarized in tables, presented in attachments as a supplementary material—Tables S1–S3. In general, most studies reported some improvements in anthropometric parameters, body composition, lipid profile, glucose metabolism, inflammatory biomarkers, blood pressure, and oxidative stress. In addition, benefits considering self‐reported physical quality of life, specific tests of memory and executive functions, and sleep quality were reported (Table S3).

### 3.4 Multidisciplinary Therapy: Main Components, Duration, and Type of Intervention

The multidisciplinary team varied from a very complete health team including physician, nurses, nutritionist, physical therapist, exercise physiologist, and psychologist to simpler models including only two health workers (e.g., nutritionist and exercise physiologist). Multidisciplinary therapy included a combination of diet, physical activity, and behavioral approaches, which was performed through different methodologies, such as behavior‐change and problem‐solving skills, sometimes also including medications. Interventions could be one‐to‐one or in groups and in different settings, for example, communities, clinics, hospitals, primary health care, and web‐based as a complementary approach [25, 67, 68, 69, 70, 71, 72, 73, 74, 75, 76, 77, 78, 79, 80, 81, 82, 83, 84, 85, 86, 87, 88, 89, 90, 91, 92, 93, 94, 95, 96, 97, 98, 99, 100, 101].

### 3.5 Effects of Multidisciplinary Therapy on Body Composition and Adipose Tissue Metabolism

Multidisciplinary therapy resulted in a reduction in body weight, body fat, and body circumferences and in preservation and/or increase of lean body mass. There were benefits in controlling metabolic syndrome, nonalcoholic fatty liver diseases, diabetes, CD, and other complications [41, 90, 102, 103, 104, 105, 106, 107, 108, 109, 110, 111].

Among older adults with obesity, we found a reduction in body weight (101.5 ± 3.8 vs. 94.5 ± 3.9 kg), BMI (36.0 ± 1.7 vs. 33.5 ± 1.7 kg/m^2^), and waist circumference (116 ± 3 vs. 109 ± 3 cm) with a 1‐year lifestyle intervention at 30‐month follow‐up with no change in fat‐free mass. Regarding short‐term multidisciplinary interventions for older and younger adults with obesity, we observed a reduction in body weight (old −3.8%, young −4.3%), BMI (old −3.9%, young −4.4%), and waist circumference (old −3.4%, young −4.1%) [74]. A prospective multicenter study reported an accentuated reduction in body weight of 19.6 kg in females and 26.0 kg in males [110]. Another intervention found a decrease of 11 cm in waist circumference, leading to a reduced prevalence of metabolic syndrome and hypertension [102]. Moreover, a family‐based nutritional intervention observed a reduction in body weight (77.3 ± 16.9 vs. 76.5 ± 16.9) after 6 months of intervention [104]. Interestingly, according to some of the extracted data, a modest (1 kg) body weight decrease was sufficient to ameliorate biomarkers of total and ectopic fat, glycemia homeostasis, and insulin resistance in patients with obesity at risk of diabetes [100].

**FIGURE 2:**
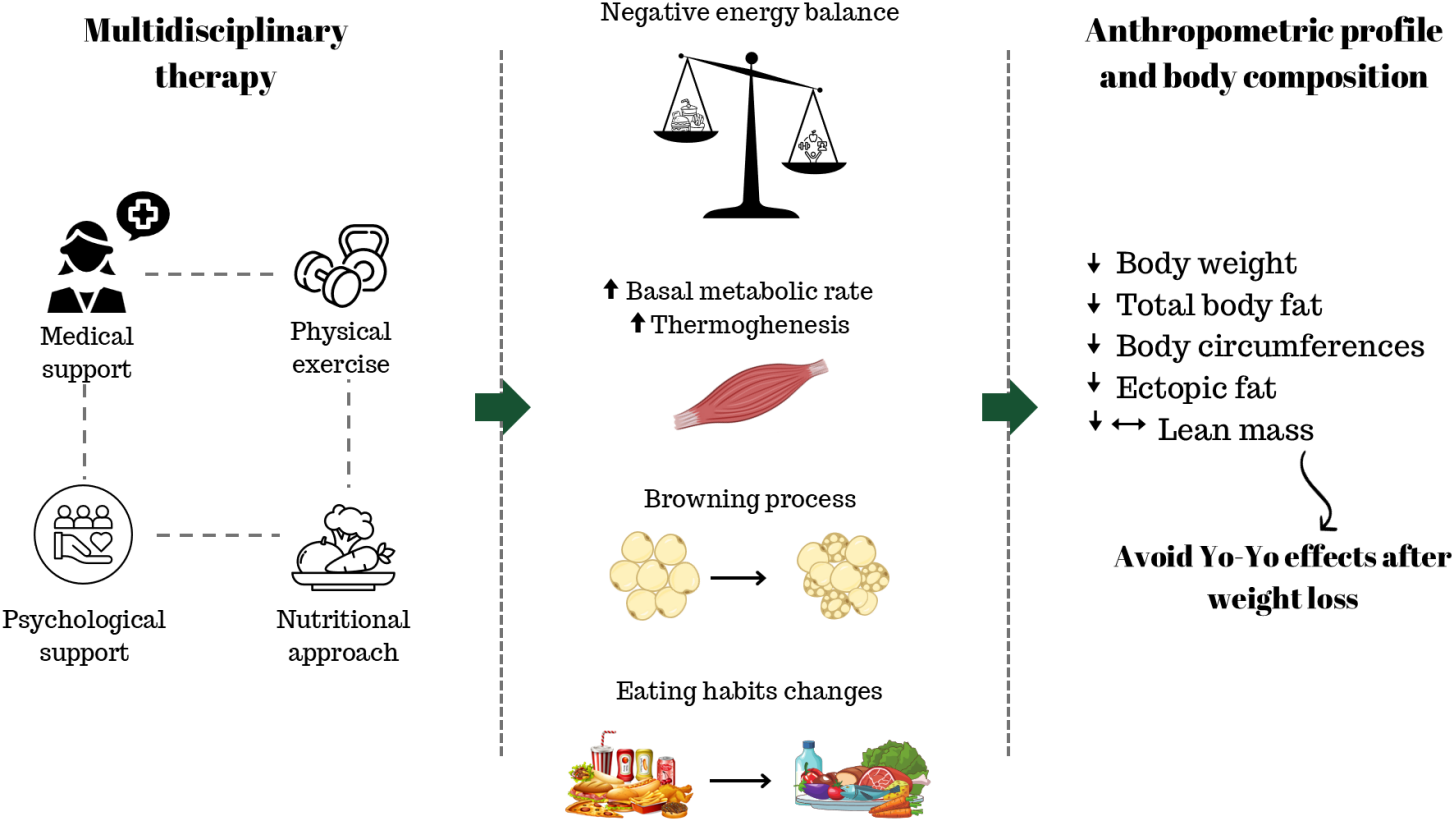
Effects of multidisciplinary therapy on body composition and adipose tissue metabolism in adults with obesity.

In general, the suggested mechanisms associating the potential impact of multidisciplinary therapy involve these pathways: (1) Nutritional approaches promote a negative energy balance and accentuated reduction in whole‐body fat [112, 113]; and (2) exercise may preserve and/or increase the lean body mass, favoring the upregulation of the basal metabolic rate; this may activate thermogenesis and *browning*, avoiding the yo‐yo effect [62, 114] (Figure 2). In addition, nutritional counseling and exercise together can attenuate modifiable risk factors, including endothelial function, cardiometabolic risks, mood, and eating disorders [115, 116]. Minor additional metabolic benefits were found for high‐intensity compared with moderate‐intensity training [117].

Activating brown adipose tissue and the conversion of white adipose tissue into brown adipose tissue, a process known as browning, using different types of exercise [118, 119, 120, 121, 122, 123, 124, 125, 126, 127] and nutritional interventions [128, 129, 130, 131, 132, 133, 134, 135], may improve thermogenesis, stimulating weight loss and ameliorating altered metabolic conditions [133, 136, 137, 138, 139, 140]. Specifically in response to exercise, different factors are activated, including the SNS, PGC1‐alpha, fibronectin type III domain–containing protein 5 (FNDC5), irisin, catecholamine's, IL‐6, meteorin‐like protein, and fibroblast growth factor 21 (FGF‐21) [119, 120, 121, 122, 123, 124, 125]. Activating the thermogenic response with different strategies leads the brown adipose tissue to increase secretion and action of uncoupling protein 1 (UCP1) in the mitochondrial inner membrane, upregulating the thermogenesis [141].

### 3.6 Effects of Multidisciplinary Therapy on Inflammatory State

Severe obesity (BMI > 45 kg/m^2^) was strongly associated with the expansion of total and visceral adipose tissue, which secretes a variety of proinflammatory adipokines, leading to the higher metabolic disturbances, such as chronic low‐grade inflammation, linking dyslipidemia, diabetes, hypertension, metabolic syndrome, and nonalcoholic fatty liver disease, thereby enhancing the risk of CD [142, 143, 144].

Adipose tissue contains several types of cells, including adipocytes, preadipocytes, fibroblasts, endothelial, and immune cells. Visceral adipose tissue secretes various bioactive peptides, called adipocytokines or adipokines, which are able to modulate several processes in the human body, including energy balance, metabolism, and inflammation [145]. Adipocytes and infiltrating macrophages evolved in the regulated inflammatory signaling cascades in different pathways; they contribute to a persistent inflammatory and prothrombotic environment in obesity through the secretion of cytokines, commonly termed adipokines [146].

The proposed mechanisms linking severe obesity with inflammation may include higher adiposity, leading to insulin resistance and increased secretion of a milieu of proinflammatory adipokines, such as leptin, interleukin 6 (IL‐6), plasminogen activator inhibitor 1 (PAI‐1), C‐reactive protein (CRP), and tumor necrosis factor alpha (TNF‐α). Conversely, in severe obesity, adiponectin, one of the most important anti‐inflammatory adipokines secreted by adipose tissue, was reduced [146, 147, 148, 149, 150]. Adiponectin concentrations decrease in people with obesity and exhibited an inverse relationship with visceral adiposity. This clinical condition further exacerbates metabolic dysfunction. Although the restoration of its normal level is important to protect against CD and nonalcoholic fatty liver diseases, both coexisting with obesity [150, 151].

C‐reactive protein, TNF‐α, and IL‐6 have been found to destroy pancreatic beta cells, thereby compromising insulin production. Adiponectin, however, plays a protective role in beta cells, reversing the damage and impaired insulin secretion induced by obesity. Additionally, adiponectin can induce anti‐inflammatory effects by increasing IL‐10 and modulating the metabolism of macrophages in humans [148, 152]. The anti‐inflammatory target in obesity is indeed mediated by a decrease in the proinflammatory hyperleptinemia state when people lose approximately 10% of their body weight [62, 153].

FGF21 has a key action in mediating inflammation in obesity. Different organs, including adipose tissue, which exert endocrine and paracrine mechanisms regulating whole‐body homeostasis, including improvements in insulin sensitivity mediated by adiponectin [146, 148], secrete this growth factor. Additional anti‐inflammatory effects mediated by omentin‐1 can inhibit the expression of lipopolysaccharide (LPS)‐induced inflammatory factors in macrophages. Moreover, angiotensin and bone morphogenetic protein 7 (BMP7) also exert anti‐inflammatory effects by inhibiting other signaling pathways, including p38 and p44/42 MAPK [150].

As observed in the analyzed studies, multidisciplinary therapy promoted clinical control in some proinflammatory adipokines and biomarkers of cardiometabolic risks, such as cardiovascular fitness, glucose metabolism, insulin‐like growth factor 1 (IGF‐1), blood pressure, LDL‐cholesterol, high‐density lipoprotein (HDL) cholesterol, triglyceride levels, Il‐6, PAI‐1, leptin, CRP, ICAM‐1, VCAM‐1, and tumor necrosis factor receptor 1 [72, 85, 99, 107, 154, 155, 156, 157].

**FIGURE 3:**
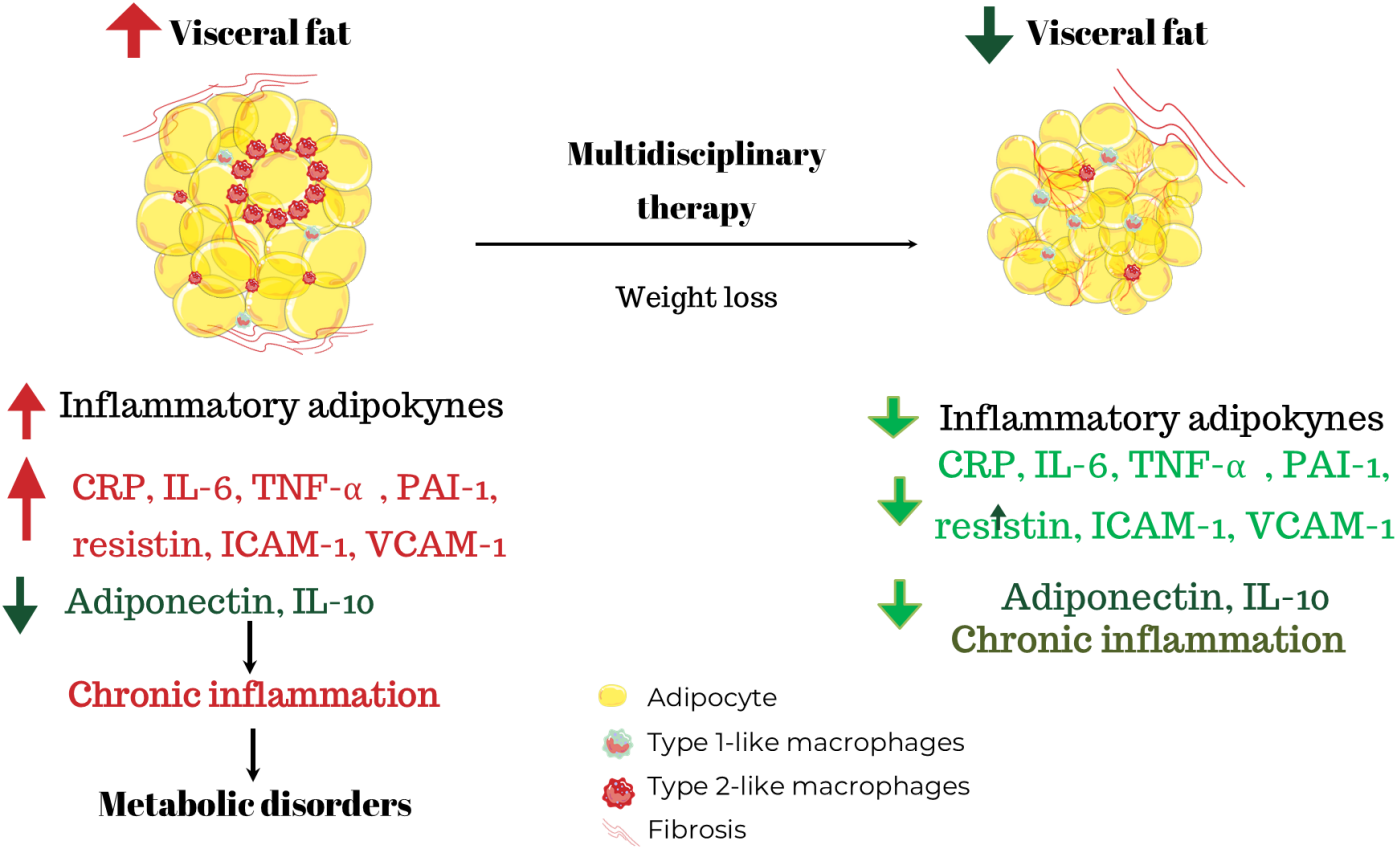
Effects of multidisciplinary therapy on inflammatory profile in adults with obesity.

In this sense, after long‐term (26 weeks) interdisciplinary therapy with lifestyle changes to treat obesity in women, an improvement of inflammatory state and cardiometabolic risk was observed with a reduction in PAI‐1 (9.95 ± 2.52 vs. 9.84 ± 2.74 ng/dL), leptin (25.60 ± 7.03 vs. 22.33 ± 7.09 mg/dL), CRP (33.61 ± 13 vs. 30.25 ± 13.72 mg/dL), ICAM‐1 (101.35 ± 38.99 vs. 60.69 ± 19.33), and VCAM‐1 (718.69 ± 322.56 vs. 399.31 ± 136.64) [107]. Evidence from a clinical trial and a systematic review and meta‐analysis indicates that physical exercise and dietary interventions together are more effective in reducing leptin and increasing adiponectin concentrations than exercise alone in individuals with obesity [158, 159]. Interestingly, hyperleptinemia state is related to a proinflammatory condition, but when this hormone is normalized after weight loss promoted by multidisciplinary therapy, this mechanism plays an essential role to sustain weight loss, favoring an increase in adiponectin concentration [85, 99, 107, 143, 149] (Figure 3).

### 3.7 Effects of Multidisciplinary Therapy on Obesity and Its Complications

A comprehensive multidisciplinary lifestyle therapy, addressing all aspects of lifestyle and risk factor management, is truly necessary to prevent and combat altogether obesity and its constellation of complications, because it is a public health challenge [14, 84, 160, 161, 162, 163, 164, 165, 166].

Multidisciplinary therapy, including clinical, nutritional, behavioral, and exercise interventions in short and long term, delivered in both nonintensive and intensive formats, has shown effectiveness in obesity management. It promotes reductions in body weight (2.8% to 29.6%), total body fat (up to 20%), and visceral/ectopic fat indirectly assessed by waist circumference, with a reduction of 1.5% to 8.8% [71, 74, 81, 84, 86, 91, 97, 100, 167, 168]. It also decreases the prevalence of metabolic syndrome (from 4% to 50%) [94, 97, 169]. Notably, 10‐year cardiovascular risk assessed by the Framingham score was reduced by 38% in just 13 weeks [97]. Improvements were also observed in several cardiometabolic parameters such as waist circumference (2.54% to 16.8%), systolic (4.66% to 9%), and diastolic blood pressure (6.4% to 12%); plasma glucose (7% to 36%), insulin (22.44% to 53%), HOMA‐IR (22.7% to 44%), HbA1c (7% to 31.93%), triglycerides (4.07% to 40%), and total cholesterol (6% to 28%) [7, 25, 68, 71, 72, 78, 80, 83, 84, 85, 93, 94, 95, 97, 101, 167, 168, 170, 171]. Some disorders such as diabetes (reduced up to 58%) [172]; sleep disorders (improves 6.6% in the sleep efficiency; 53% in the snoring, and 56.6% lower risk of developing OSA) [73, 79, 89, 91]; bone diseases (increase of 12% in lumbar spine BMC, 6% in lumbar spine BMD, and 5% in femoral neck BMD) [75]; polycystic ovary syndrome (PCOS) (improves from 10–15% in the emotion score; from 9% to 18% in the infertility problem score; and from 17% and 24% in the menstrual problems score) [88, 96]; asthma symptoms (increase of 68.6% in symptom‐free days compared with the control group) [79]; and cancer (reduce 7% in body weight; 6.5% in BMI; 9.5% in body fat; 13.5% in TC/HDL ratio; and 8.4% in triglycerides) [92].

Metabolic syndrome (MetS) is a major health problem worldwide and the main risk factor for metabolic‐associated fatty liver disease (MAFLD). Nonalcoholic fatty liver disease (NAFLD) is the most prevalent cause of chronic liver morbidity and is associated with obesity, diabetes, and metabolic syndrome. Evidence points out that NAFLD is a hepatic manifestation of metabolic syndrome because this disease coexists with obesity, insulin resistance, dyslipidemia, and type 2 diabetes [67, 173].

Hohenester et al. [84] found that in patients with diagnosis of obesity and nonalcoholic steatohepatitis (NASH), weight reduction > 10% was favorable in severe obesity control, including a significant reduction in the prevalence of abnormal serum transaminases from 81.0% to 50.5%, and in the NAFLD fibrosis score, from 11.8% to 0%. Additionally, Haufe et al. [83] investigated the effect of a telemonitoring supported intervention on liver parameters of inflammation and fibrosis in individuals with metabolic syndrome. Authors found that after 6 months of intervention, ALT and AST enzyme concentrations significantly decreased compared with the control group. In addition, 31 patients (33%) had normal ALT levels in the intervention group compared with 15 patients (14%) in the control group.

Among the comorbidities related to obesity, CD was the first cause of death and disability globally, while type 2 diabetes remained 2nd in this association [97]. Multiple mechanisms are proposed linking obesity to CD development. First, the accumulation of fatty acid within the myocardium can impair cardiac contractility, promoting left ventricular dysfunction. Second, obesity may disrupt the renin–angiotensin–aldosterone system (RAAS), leading to an increase in sodium retention and an increase in blood pressure, predisposing people with obesity to coexist with hypertension [151]. Third, obstructive sleep apnea (OSA), a breathing disorder correlated with metabolic syndrome and increased cardiovascular risk, is highly prevalent in people with obesity [79, 174]. Multidisciplinary therapy presents great potential because it confers a multitool approach, targeting a diversity of related risks coexisting with obesity, and is useful to prevention, monitoring, and management of these diseases [11, 70, 97, 175].

The main purpose mechanisms linking obesity with its complications is related to the low‐grade inflammation mainly caused by the adipocytes hypertrophy, in particular those located in the visceral depot [176]. The inflammatory nature of obesity has become a key factor to explain its role linking many chronic diseases, including the development of CDs, metabolic syndrome, OSA, asthma, NAFLD, diabetes, and hypertension. People with obesity showed altered plasma concentrations of proinflammatory cytokines, such as resistin, visfatin, IL‐6, IL‐18, TNF‐α, and CRP, which together accentuate the proinflammatory milieu commonly associated with obesity [39, 79, 146, 151, 174, 176].

**FIGURE 4:**
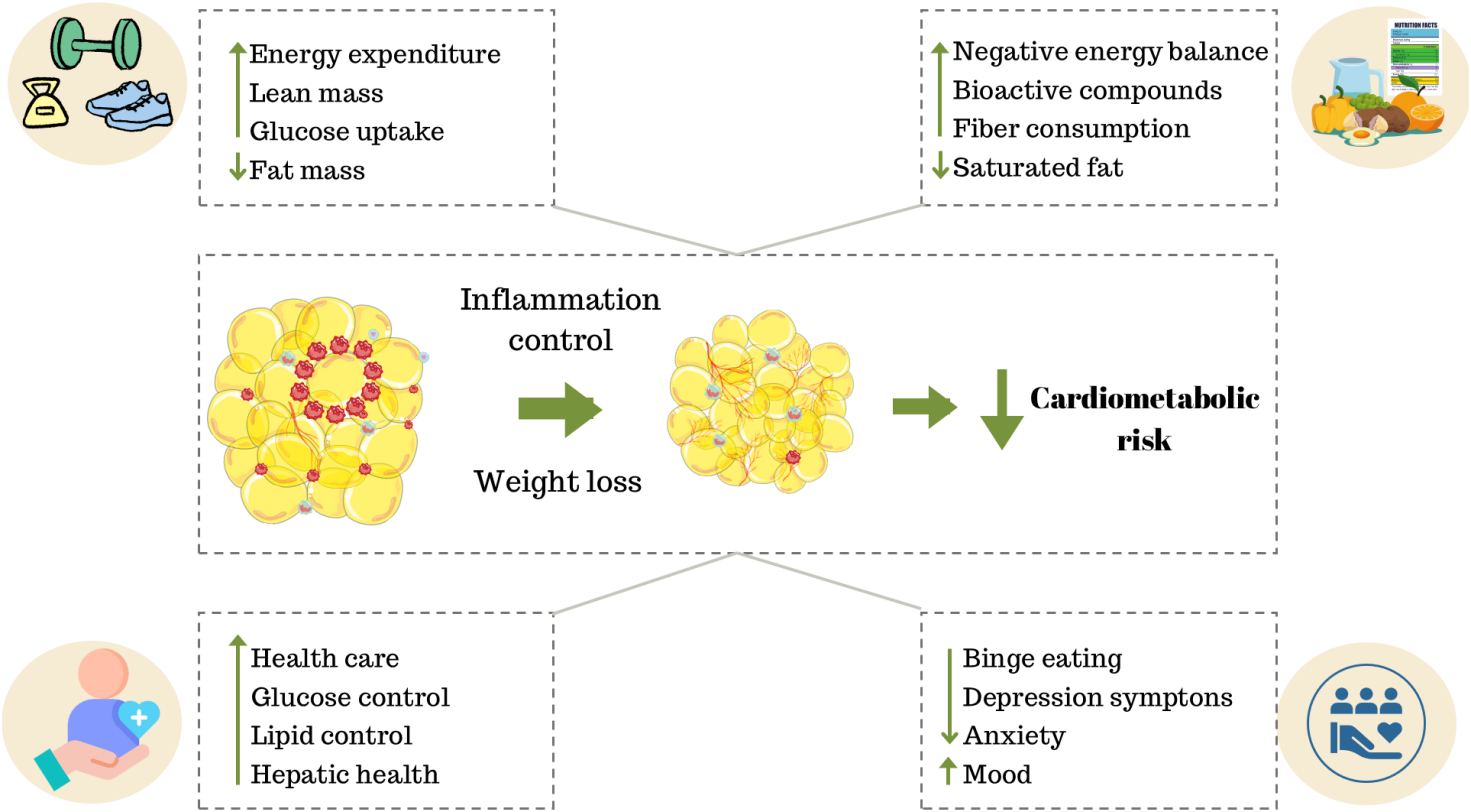
Effects of multidisciplinary therapy on its complications in adults with obesity.

The potential impact of multidisciplinary therapy on obesity and its complications, both the in short term and long term, is mediated for a wide range of outcomes, including (1) *nutritional therapy* contributes not only to energy balance leading to weight loss but also to improvements in the pool of bioactive compounds that might act as co‐adjuvants to decrease inflammation [11, 71]; (2) *Exercise therapy* increases lean body mass, energy expenditure, metabolic rate, and browning of white adipose tissue; exercise also leads to improvements in glucose uptake/insulin sensitivity, adiponectin, and leptin concentrations, downregulating the inflammatory state [112, 113, 141]; (3) *Behavioral change* has the potential to ameliorate depression and anxiety symptoms, reduce binge eating and improve quality of life [79, 96]; (4) *Clinical support* and counseling favors the appropriate use of medication and increases adherence to multidisciplinary interventions [177, 178] (Figure 4).

### 3.8 Effects of Multidisciplinary Therapy on Behavior and Quality of Life

**FIGURE 5:**
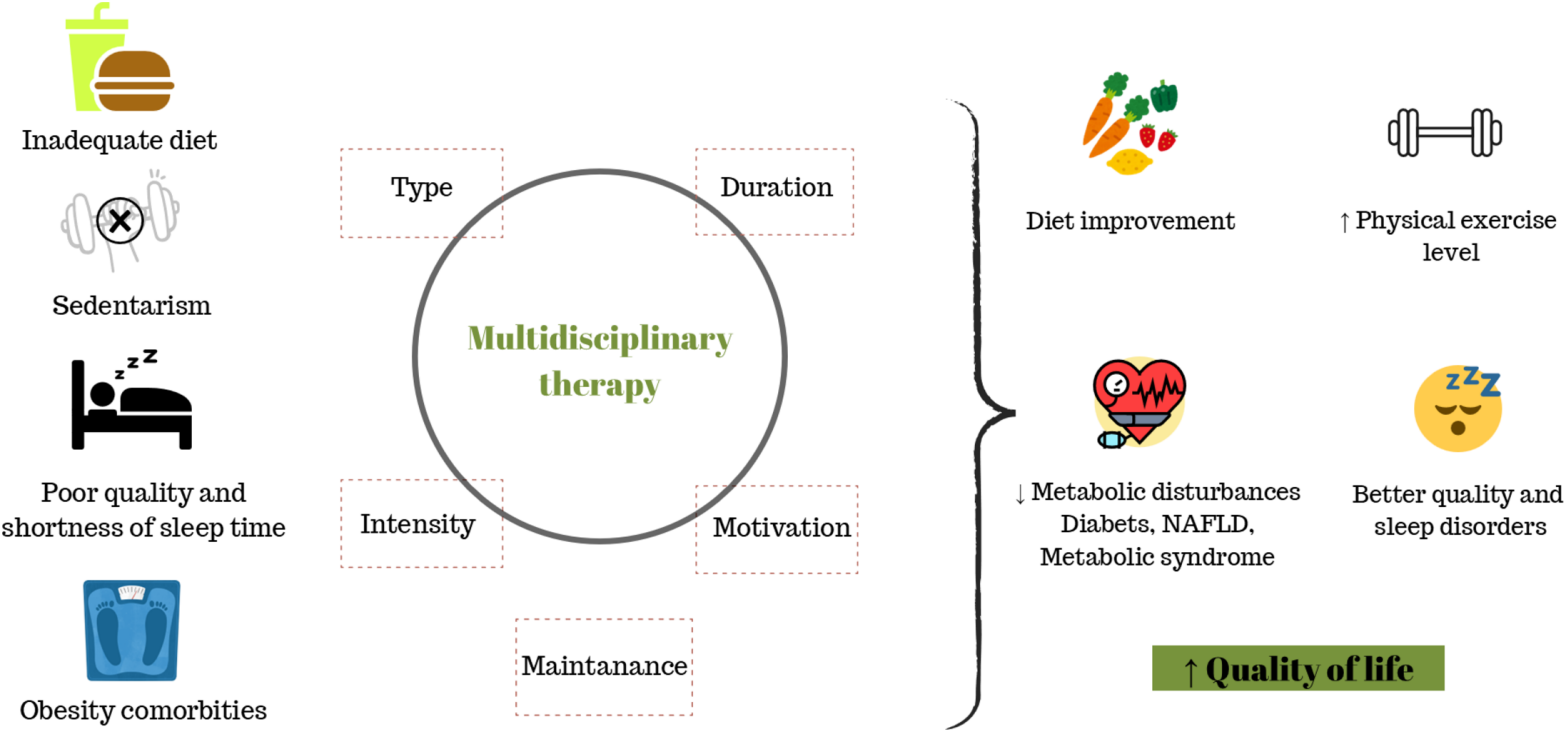
Effects of multidisciplinary therapy on behavior and quality of life.

Lifestyle approaches need to consider different cognitive profiles in order to facilitate and/or impair long‐term effects of therapy and its maintenance, behavior changes, and mood disorders, including depressive symptoms [64, 74, 79, 94, 96, 112, 115, 179, 180, 181] (Figure 5).

A recent study set out to determine if different variants in genes previously associated with metabolic syndrome may influence the magnitude of change in metabolic syndrome risk during a 1‐year lifestyle intervention. Authors found that individuals with certain genotypes might benefit more from a lifestyle intervention, suggesting the nutrigenomic as part of metabolic syndrome treatment [11]. Changes in the genes related to intermediate metabolism, diabetes, inflammation, and signal transduction might have potential benefits in health, reducing cardiovascular risk factors [182].

A study on intensive inpatient multidimensional therapy, including clinical support, nutritional counseling (once a week individually), physical exercise (2 h/day in the morning and afternoon; every day), and behavioral counseling (once a week individually), targeting adult and elderly patients with severe obesity (BMI > 45 kg/m^2^) evaluates the short‐term effectiveness of this approach. Regarding diet, younger patients presented improvement (31.8 ± 8.3 vs. 27.5 ± 7.8; *p* < 0.001) compared with older patients (26.3 ± 7.2 vs. 25.9 ± 6.2; *p* > 0.05). In addition, considering health‐related quality of life (HRQOL), younger patients showed improvement in the three dimensions of the analyzed psychometric scales (physical, mental, and total score in the 36‐item Health Survey) while older patients showed benefits in the physical dimension (46.4 ± 21.5 vs. 53.0 ± 22; *p* = 0.027) and mental dimension (53.1 ± 20.2 vs. 63.2 ± 16.5; *p* = 0.018). Results suggest that application of intervention in the long term may accentuate the beneficial impact in quality of life through better management of obesity‐associated morbidities and reduced disabilities [75].

Another emerging type of approach is web‐based physical activity and nutritional behavioral changing interventions [183, 184]. Recent studies have found an association of these interventions with a reduction in cholesterol and caloric intake as well as improvements in physical activity. There were additional benefits on eating disorders and HRQOL [168, 185].

### 3.9 Multidisciplinary Therapy as Support to Medication and Bariatric Surgery

When lifestyle and behavior approaches fail or do not sufficiently reduce body weight [161], medications and/or bariatric surgery may be required [23, 31, 38, 41, 47, 54, 186].

**FIGURE 6:**
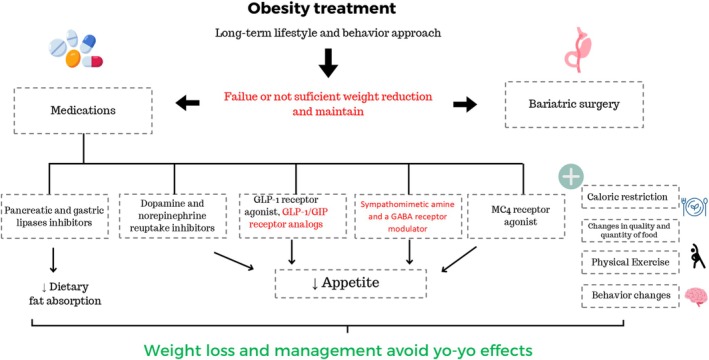
Effects of medications in weight management in adults with obesity.

Medications may be recommended as an adjunct to a comprehensive multidisciplinary weight loss program of diet, exercise, and behavior therapy. Liraglutide and semaglutide *plus* an intensive behavioral therapy are more effective than placebo plus intensive behavioral therapy in promoting clinical reduction of body weight [186, 187]. Notwithstanding, the combined effects of sibutramine *plus* lifestyle intervention are more effective than either type of isolated applied approach as a single therapy in promoting weight loss, thus reinforcing the importance of multidisciplinary therapy in obesity management [187] (Figure 6). Weight loss maintenance may be hampered by adaptive biological responses, that is, increase in hunger or reduction in lipid oxidation [41]. Multidisciplinary therapy may aid patients in improving diet, exercise practices, and medication adherence, which may prevent yo‐yo effects [26, 28, 62, 188].

A randomized, double blind, placebo‐controlled clinical trial assessed the effect of Liraglutide and multidisciplinary lifestyle intervention on visceral adipose tissue reduction measured with magnetic resonance images (MRI) in adults with obesity and metabolic syndrome and high CD risk. Authors reported a reduction in visceral adipose tissue, which may explain the benefits of Liraglutide on cardiovascular outcomes [30]. Previous work reported that weight loss from Liraglutide use (3%–4%) was associated with a reduction of atherothrombosis risk in women with obesity [88].

Multidisciplinary therapy, using exercise, nutritional and behavioral changes, plays an important role before bariatric surgery by improving cardiometabolic risk factors and favoring weight loss maintenance [189, 190, 191, 192, 193]. Aakre and colleagues [54] reported benefits of bariatric surgery compared with intensive lifestyle intervention on risk for CDs. They found that BMI was reduced by 14.4 kg/m^2^ versus 3.9 kg/m^2^ in the bariatric surgery compared and the intensive lifestyle intervention group, respectively. Cardiac troponins decreased after bariatric surgery (*p* = 0.014 [cardiac troponin T], *p* = 0.065 [cardiac troponin I]) and increased in those treated with lifestyle intervention (*p* ≤ 0.021). CRP decreased in both groups.

Bariatric surgery promotes long‐lasting accentuated weight loss, favoring the control of many altered clinical parameters, including proinflammatory, endothelial, and thrombotic biomarkers (TNF‐alpha, IL‐1B, IL‐6, PAI‐1, C‐reactive protein, intercellular adhesion molecule‐1 [ICAM‐1], leptin, resistin, and leptin/adiponectin ratio). Conversely, anti‐inflammatory biomarkers, adiponectin and interleukin‐10 (IL‐10) were increased in individuals with extreme obesity after bariatric surgery in both short‐ (6 months) and long‐term (2 years) follow‐up. They found that in patients with severe obesity who have hyperleptinemia, there were higher circulating PAI‐1 levels, which may increase the risk for CD. In fact, changes in leptin concentrations were associated with decreased PAI‐1 levels [27, 53, 57, 188, 194].

Farias and colleagues [27] reported that BMI and excess body weight had decreased by 15.79 ± 1.21 kg/m^2^ (*p* < 0.01) and 83.80 ± 24.50%, respectively, after 2 years of follow‐up postbariatric surgery. There were reductions in leptin (38.00 vs. 4.87 ng/mL), CRP (22.84 vs. 4.20 μg/mL), and PAI‐1 (28.72 vs. 20.46 pg/mL) concentrations as well as increased anti‐inflammatory biomarkers such as adiponectin (6.82 vs. 27.13 μg/mL) and the adiponectin/leptin ratio (0.18 vs. 5.42).

To sustain long‐term weight loss management after Roux‐en‐Y gastric bypass (RYGB) (2 years), the suggested mechanisms include eating behavior, neural and peripheral signals of satiety, gut microbiota, and an intrinsic activation of anorexigenic pathways, in which leptin upregulates alpha‐MSH. This anorexigenic neuropeptide stimulates lipolysis and browning of adipose tissue, which increase metabolic rate and thermogenesis, avoiding the compensatory hormonal response that occurs after accentuated weight loss, which may lead to the reduction in energy expenditure and, consequently, weight regain [27, 28, 29, 39, 195].

Authors also found an increase in circulating neuropeptide α‐melanocyte‐stimulating hormone (α‐MSH), activating anorexigenic pathway and promoting a reduction in food intake after long‐term follow‐up RYGB (2 years). Additionally, the authors suggest other mechanisms, such as the impact of augmented PYY and glucagon‐like peptides (GLP‐1 and GLP‐2) 2 years after RYGB, which enhance satiety in the long term, favoring the neuroendocrine regulation of energy balance. Furthermore, leptin levels were decreased (38.0 ± 4.0 vs. 4.9 ± 1.5, *p* < 0.001) and adiponectin levels were increased (6.8 ± 0.6 vs. 27.1 ± 4.1, *p* < 0.001) after long‐term follow‐up [26].

The reduction of inflammatory state and improved insulin and leptin sensitivity favor the action of α‐MSH and PYY on appetite control, increased satiety, and energy expenditure after RYGB, because both stimulate the anorexigenic pathways in a long‐term RYGB, thus promoting the effectivity of the procedure to induce sustained weight loss. Additionally, the increase in adiponectin concentrations after RYGB accentuates its anti‐inflammatory effects, improving insulin sensitivity and reducing the prevalence of cardiometabolic risk factors [26].

**FIGURE 7:**
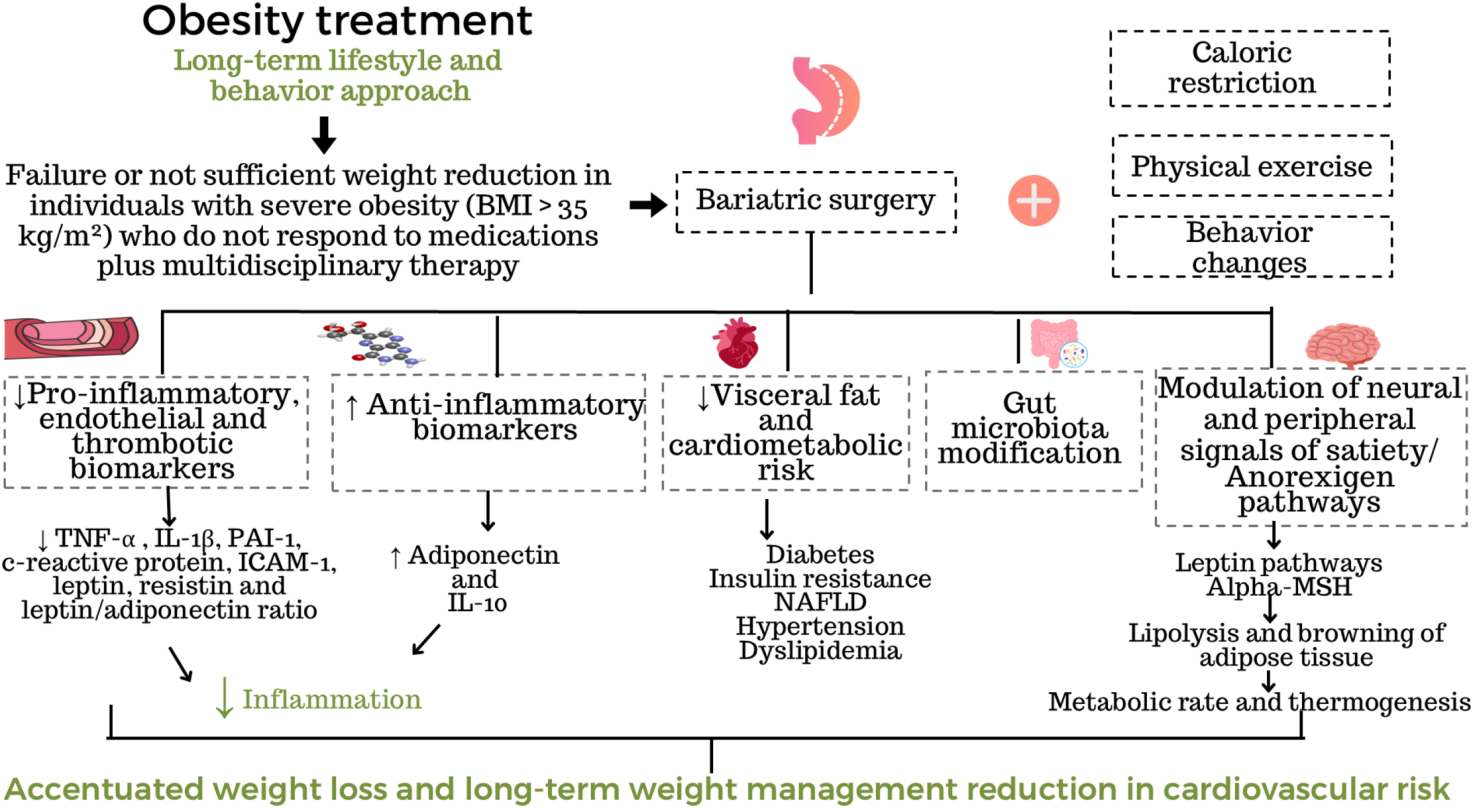
Effects of bariatric surgery in weight loss and reduction in cardiovascular risk in adults with obesity.

Importantly, lifestyle changes followed by bariatric surgery promote lower inflammatory and thrombotic biomarkers, including (high‐sensitivity C‐reactive protein, tumor necrosis factor‐α, interferon‐γ, interleukin‐1 receptor antagonist, IL‐6, and IL‐13, leptin, insulin, fibrinogen, and plasminogen activator inhibitor‐1). These risk factors were normalized, 1 year after surgery. There was a positive correlation between changes in C3 and body mass index, body weight, coagulation parameters, inflammatory parameters, and leptin, respectively, showing the associated impact of both health approaches [60] (Figure 7).

## 4 Discussion

We have found that multidisciplinary therapy targeting obesity, weight loss, visceral fat, and the whole‐body fat may play an important role in the first line of obesity management programs [14, 47, 84, 160, 161, 162, 163, 164, 165, 166].

The main mechanisms linking obesity and its complications are related to the low‐grade inflammation mainly caused by adipocyte hypertrophy, particularly those in the visceral depot [196]. Multidisciplinary therapy promotes continuous weight loss processes by multidimensional factors including improvement in food patterns, an increase in physical activity levels, and better management of mood disorders and control of clinical disorders.

Effectiveness will depend on intervention types, duration, intensity, and adherence [39, 85, 87, 102, 186, 197, 198, 199, 200, 201]. Lifestyle approaches need to consider the role of families, the community, the environment, and different cognitive profiles in order to foster long‐term intervention implementation and sustained beneficial effects. An efficient multidimensional approach to reduce modifiable risk factors for targeting the multimorbidity associated with obesity is required [201].

In this context, the concept of maximum weight attained in life (MWAL) emphasizes the clinical benefits of modest weight reduction as opposed to achieving a “normal” BMI. Evidence shows that a weight loss of at least 5% already promotes relevant health improvements, whereas losses of 10%–15% bring even greater benefits, regardless of final BMI. Authors are encouraged to consider whether individuals can be classified as having “reduced obesity” (5%–10% weight loss) or “controlled obesity” (> 10%–15% weight loss). This proposal, endorsed by the Brazilian Society of Endocrinology and Metabolism (SBEM) and the Brazilian Society for the Study of Obesity and Metabolic Syndrome (ABESO), does not replace other classifications but serves as a complementary tool that highlights the importance of weight maintenance and clinical progress over absolute BMI normalization [202]. Our review evidence found that a reduction above > 10% in body weight in patients with obesity and nonalcoholic steatohepatitis has favorable benefits in severe obesity control [84]. Additionally, a multidisciplinary lifestyle approach with a ≥ 10% decrease in body weight may reduce cardiovascular risk factors associated with obesity [7].

While several previous reviews have addressed multidisciplinary therapy in obesity [203, 204, 205, 206], we provide a more systematic and comprehensive literature search that included studies published up to 2025, allowing us to integrate the most recent evidence. We emphasize the interaction between nutritional and psychological counseling, exercise training, and the use of medications and bariatric surgery when improvements in lifestyle are not sufficient to control obesity and its complications. Regarding the latter, we highlight how the combination of these strategies provides synergistic benefits beyond what has been described individually. Lastly, our review presented mechanisms of action of effects of multidisciplinary therapy on short‐ and long‐term outcomes, for example, biomarkers of inflammation, cardiometabolic risk factors, dyslipidemia, diabetes, hypertension, metabolic syndrome, nonalcoholic fatty liver diseases, atherosclerosis, CD, obstructive sleep apnea, and psychosocial ameliorations.

The strengths of this review include the multiple sources that were searched and the detailed description of many related outcomes and mechanisms involved in the types of multidisciplinary therapies. The main limitation was the wide diversity of clinical designs and the lack of follow‐ups in most studies, which precluded us from completing meta‐analyses.

Regarding implications, multidisciplinary therapy may continue prioritized as a strategy to manage obesity; also to prevent and control complications in adults. Clinical guidelines should provide further guidance regarding the health workers required, detailed intervention protocols, and when further interventions, for example, pharmacotherapy, may be required. Future research should investigate in deeper detail the potential impact of different clinical approaches and report longer follow‐up periods.

## 5 Conclusion

Obesity as a chronic and multifactorial disease requires a multidisciplinary team acting together in a holistic multitarget intervention. Nutrition, exercise, and behavioral approaches are commonly used together as the first line of interventions. When this is insufficient, patients may need complementary clinical support including medications and bariatric surgery. Multidisciplinary therapy should aim for a reduction of fat mass associated with a preservation or increase in lean body mass and a control of proinflammatory state, thus preventing or controlling obesity complications and improving quality of life.

## Author Contributions

Conceptualization of the study: Ana Raimunda Dâmaso. Responsible for the writing of the original draft: Ana Raimunda Dâmaso, Flávia Campos Corgosinho, Deborah Cristina Landi Masquio, Raquel Munhoz da Silveira Campos. Acquisition, analysis, or interpretation of data: Ana Raimunda Dâmaso, Flávia Campos Corgosinho, Deborah Cristina Landi Masquio, Raquel Munhoz da Silveira Campos, Fabiana Kattah, Nayra Figueiredo. Reviewing and editing the manuscript: Ana Raimunda Dâmaso, Cintia Cercato, Lian Tock. Supervision: Ana Raimunda Dâmaso.

## Funding

This study was supported by the Department of Nutrition and Food Safety at the World Health Organization (WHO), with additional financial support from the Norwegian Agency for Development Cooperation (NORAD), the Swedish International Development Cooperation Agency (SIDA), the Government of the Grand Duchy of Luxembourg, and the Government of Germany (BMG).

## Conflicts of Interest

The authors declare no conflicts of interest.

## Supporting information


**Table S1:** Population characteristics and variables analyzed in the selected studies
**Table S2A:** Multidisciplinary therapies reported in the selected studies
**Table S2B:** Description of different approaches of multidisciplinary interventions
**Table S3:** Effect of multidisciplinary treatment among adults of different populations

## Data Availability

The data that support the findings of this study are available in the Supporting Information of this article.
