# Supplementary material for: Multidisciplinary Therapy to Target Obesity and Its Complications in Adult Population: A Narrative Review
Source: Obes Rev. 2026 Jan 15;27(7):e70093. doi: 10.1111/obr.70093 (PMC13243351; doi:10.1111/obr.70093)
Supplement: Supplementary file 1 — Table S1: Population characteristics and variables analyzed in the selected studies Table S2A: Multidisciplinary therapies reported in the selected studies Table S2B: Description of different approaches of multidisciplinary interventions Table S3: Effect of multidisciplinary treatment among adults of different populations [file OBR-27-e70093-s001.docx]

**Supplementary Materials**

**Multidisciplinary Therapy to Target Obesity and its Complications in Adult Population: A Narrative Review**

Ana Raimunda Dâmaso, PhD^1,2,3^; Flávia Campos Corgosinho, PhD^4,5^; Deborah Cristina Landi Masquio, PhD^1,6^; Nayra Figueiredo^5^; Fabiana Kattah^4^; Cintia Cercato, MD-PhD^2,7^; Lian Tock; MD^1,2^; Raquel Munhoz da Silveira Campos, PhD^1,8^

*1 Post-Graduate Program of Nutrition - Federal University of São Paulo - Paulista Medicine School - UNIFESP-EPM*

*2 Brazilian Association of Study on Obesity and Metabolic Syndrome - ABESO*

*3 National Council for Scientific and Technological Development - CNPq*

*4 Post-Graduate Program in Nutrition and Health - Nutrition Faculty - Federal University of Goiás - UFG*

*5 Post-Graduate Program in Science and Health - Medicine Faculty - Federal University of Goiás - UFG*

*6 Post-Graduate Program of Professional Nutrition: from birth to adolescence - Centro Universitário São Camilo*

*7 Post-Graduate Program of Endocrinology - University of São Paulo - Medicine School - FMUSP*

*8 Post-Graduate Program of Interdisciplinary in Health Sciences - Federal University of São Paulo - Campus Baixada Santista – UNIFESP-CBS*

***Corresponding author***

Dra. Raquel Munhoz da Silveira Campos

Post-Graduate Program of Interdisciplinary in Health Sciences - Federal University of São Paulo - Campus Baixada Santista – UNIFESP-CBS. XV de Novembro, 195, 5^th^ floor – Santos/São Paulo - Brazil

E-mail: [raquel.munhoz@unifesp.br](mailto:raquel.munhoz@unifesp.br)

**Table S1.** Population characteristics and variables analyzed in the selected studies

| **Authors** | **Country** | **Study design** | **Sample size** | **Age (years)** | **Variables analysed** |
| --- | --- | --- | --- | --- | --- |
| Esposito et al.  2003 | Italy | Randomized  Single-blind trial | 120 women | 20 - 46 | Weight, waist-hip ratio, HOMA-IR, serum total and HDL-c, triglyceride and glucose levels; plasma free fatty acids (FFAs), cytokine (IL-6, IL-18); hs-CRP; adiponectin; 3-day food intake. |
| Womble et al., 2004 | United States | Randomized controlled trial | 47 women | 18-65 | Weight; blood pressure; triglycerides; glucose, total cholesterol ; HDL-c; LDL-c; Beck Depression Inventory-II; Medical Outcomes Study, Short Form-36 Health Survey; Eating Inventory. |
| O’Brien et al.  2006 | Australia | Randomized, controlled trial | 80 patients  (women=61, men=19) | 20 - 50 | Weight; fasting blood glucose level; serum insulin level; QUICKI; and a lipid profile, including HDL-c and LDL-c levels, presence of the MetS and quality of life (36-Item Short Form Health Survey. |
| Tuthill et al.  2007 | Ireland | Prospective randomized  controlled trial | 68 individuals  (women=21, men=47) | 59 | Blood tests, anthropometric measurements, and quality of life assessment using the 36-Item Short Form Health Survey (SF-36). HOMA- IR and cardiovascular risk scores were computed using the United  Kingdom Prospective Diabetes Study (UKPDS) risk engine. |
| Hofso et al.  2010 | Norway | Controlled clinical trial | 139 individuals  (women=97, men= 42) | 19 - 66 | Weight; waist and hip circumferences; glucose and lipid metabolisms, blood pressure, albuminuria, left ventricular hypertrophy, low-grade inflammation, energy intake, and physical activity. |
| Kalter-Leibovici et al.  2010 | Israel | Open, parallel-group, randomized trial | 201 women | 35 - 54 | Fasting glucose; triglycerides, HDL-c; insulin, and hs-CRP; blood  pressure; body weight; waist circumference; leisure physical activity; 36- Item Short Form Health Survey. |
| Thomson et al.  2010 | Australia | Randomized, controlled prospective clinical intervention | 49 women | 29.3±0.7 years | Centre for Epidemiologic Studies Depression Scale (CES-D) to assess Health-related quality of life (HRQOL) and depression; polycystic ovary syndrome questionnaire (PCOSQ). |
| Straznicky et al.  2011 | Australia | Randomized, controlled trial | 18 individuals  (women=4, men=14) | 45 - 65 | Weight, body mass index, waist circumference, Indirect calorimetry, insulin, leptin, nonesterified fatty acids (NEFA), hs-CRP, plasma glucose,  triglycerides, HDL-c, systolic blood pressure, diastolic blood pressure. |
| Bischoff et al.  2012 | German | Prospective multicentre  observational study | 8296 participants (women=6111, men=2185) | 18 - 70 | Weight, waist circumference, waist-to-height ratio, blood pressure, and laboratory parameters in serum (fasting glucose, triglycerides, total cholesterol/low-density lipoprotein–cholesterol, high-density lipoprotein cholesterol, ALT, g-glutamyl-transpeptidase, sodium, potassium, creatinine, and uric acid). |
| Vetter et al.  2013 | United States | Randomized controlled trial | 390 individuals  (women=311, men=79) | ≥21 | Weight, lipids, glucose, insulin, HOMA-IR, blood pressure, lipids, and hs- CRP. |
| Waters et al.  2013 | United States | Thirty-month follow-up pilot  study of a 1-year lifestyle intervention trial | 16 individuals  (women=11, men=5) | ≥65 | Body weight and composition, physical function, markers of the metabolic syndrome, glucose and insulin response to an oral glucose tolerance test, bone mineral density, liver and renal function tests, and food diaries. |
| Donini et al.  2014 | Italy | Observational prospective cohort study | 161 individuals  (women=136, men=25) | 18 - 65 | Body weight; continuation of nutritional treatment at the time of the Follow-up (FU); justification for any abandonment of multidisciplinary obesity treatment (NPPRP) or standard nutritional treatment (SNT); possible changes in eating behaviour; performance of a structured exercise program and the self-perception of an active lifestyle; possible use of other outpatient nutrition services, beauty centres, or use of  anorectic drugs. |
| Halperin et al.  2014 | United States | Randomized, parallel-group, pragmatic trial | 39 patients  (not reported sex) | 21 - 65 | Weight; waist circumference; fasting plasma glucose; HbA1c; seated blood pressure; body composition; basal metabolic rate; 6-minute walk test; 36-Item Short Form Health Survey (SF-36); Barriers to being Physically Active; EuroQol 5 Dimensions (EQ-5D) (EuroQol Group); Problem Areas In Diabetes (PAID); and Impact of Weight on Quality of Life–Lite (IWQOL). |
| Nestvold et al.  2014 | Norway | Prospectively  Study | 97 individuals  (women=68, men=29) | >18 | Waist circumference; Complement components C3 and C4; fibrinogen;  cytokines, PAI-1, insulin, and leptin. |
| Courteix et al.  2015 | France | Randomized controlled Trial | 71 individuals  (women=39, men=31) | 50 - 70 | Bone mineral content (BMC) and density (BMD), body weight, fat mass, lean body mass, visceral fat, insulin, pro-inflammatory cytokines (TNF-α, IL-1 and IL-6), adipokines leptin and adiponectin, PAI-1, HOMA-IR, and bone metabolism markers (osteocalcin, procollagen type I N-terminal  propeptide (PINP) and type I-C telopeptide). |
| Ding et al.  2015 | United States | Randomized, parallel-group, pragmatic trial | 40 individuals  (women=18, men=22) | 21 - 65 | Weight; height; waist circumference; and seated blood pressure; Body composition; 6-minute walk test; 36-Item Short Form Health Survey (SF- 36) version 2, Barriers to Being Active; Euro-QOL 5 Dimensions (EQ-5D); Problem Areas in Diabetes (PAID); and Impact of Weight on Quality of Life (IWQOL)-Lite. The United Kingdom Prospective Diabetes Study (UKPDS) Risk Engine; HbA1c; fasting glucose. |
| Jamar et al. 2015 | Brazil | Controlled clinical trial | 30 women | 30 - 50 | Weight; waist circumference; BMI; fat-free mass; body fat; energy and macronutrients intake; total cholesterol; LDL-c; leptin; adhesion molecules ICAM-1 and VCAM-1; CRP. |
| Serrano-Ferrer et al. 2016 | France | Randomized, controlled trial | 87 individuals  (women=49, men=38) | 50 - 70 | Left ventricular regional myocardial function, epicardial adipose tissue, TNF-α, adiponectin and PAI-1, triglycerides, HDL-c, LDL-c, fasting glucose, insulin, HbA1c, HOMA-IR, hs-CRP. |
| Jahangiry et al. 2017 | Iran | Randomized controlled trial | 160 individuals with metabolic syndrome  (women=54; men=106) | Over 20 | Weight, BMI, abdominal obesity, systolic/diastolic blood pressure, fasting glucose, triglycerides, HDL-c, waist circumference, physical activity measured using the International Physical Activity Questionnaire at the last 7 days (IPAQ), food frequency measurement (Food Frequency Questionnaire (FFQ), and Health-related quality of life (HRQOL) using 36- Item Short Form Health Survey (SF-36). |
| Panosian et al. 2017 | United States | Randomized  parallel-group trial | 38 individuals  (women=23, men=15) | RYGB: 50.7 ± 7.6;  IMWM: 52.6 ±4.3 | Weight; BMI; HbA1c; 6-minute walk test (distance; oxygen saturation and heart rate); IWQOL-total and physical function; 36-Item Short Form Health Survey (SF-36) ,total and Physical Function. |
| Budui et al.  2018 | Italy | Clinical trial | 259 individuals  (women=145, men=70) | Younger  participants: 48.2±11.5;  Older participants: 69.3±3.5 | Serum levels of glucose, HbA1c, total cholesterol, HDL-c, triglycerides, and uric acid, blood pressure, use of any antihypertensive drug, abdominal ultrasound, [18]. Obstructive sleep apnoea syndrome diagnosis, weight, height, BMI, waist circumference, neck circumference, fat-free mass (kg/m), fat mass (kg/m), total body water (TBW, L/m), were calculated by taking into consideration BIA and anthropometric measurements. 24-h dietary recall and diet history, 36-Item Short Form Health Survey (SF-36), Physical Component Summary Scale (PCS), and the Mental Summary Component Scale (MCS). Binge eating was tested with the use of the Binge Eating Scale (BES), cardiopulmonary exercise test (CPET),VO2, and carbon dioxide production (VCO2) and pulmonary ventilation (VE) were measured breath-by-breath through an open-circuit gas analysis system. Functional capacity was also assessed through the Six-Minute Walk Test. |
| Castro et al.  2018 | Spain | Clinical trial | 20 individuals  (women=12, men=8) | 18 - 65 | Body weight, BMI, Body composition (dual-energy X-ray absorptiometry), bone mineral content, regional lean mass, fat-free mass, fat mass percentage (FM%), and visceral fat mass. Ketosis was determined by measuring ketone bodies, specifically β-hydroxy-butyrate (β-OHB), in capillary blood by using a portable meter. Glucose, insulin, HbA1C; Thyroid-stimulating hormone (TSH), free thyroxine (FT4), and free triiodothyronine; overnight fasting plasma levels of ghrelin and leptin; fasting plasma levels of dopamine; food cravings questionnaire (FCQs), quality of life (QoL), daytime sleepiness and sleep quality, EMAS- Sexual Function Questionnaire; the Female Sexual Function Index—FSFI; multidimensional alcohol craving scale (MACS),; Physical Activity Questionnaire (IPAQ); Epworth Daytime Sleepiness Scale (ESS); Pittsburgh Sleep Quality Index (PSQI); Impact of Weight on QoL (IWQOL- Lite©). |
| Freitas et al.  2018 | Brazil | Randomized  controlled trial | 51 individuals  (women=50, men =1) | 30 - 60 | Average number of steps per day, the time spent performing moderate  to vigorous physical activities (min/day), light-intensity physical activity, and sedentary time, cardiopulmonary exercise test; peripheral muscle strength; weight, BMI, Hospital Anxiety and Depression Scale (HADS), number of asthma symptom–free days; The Berlin Questionnaire; sleep quality. |
| Gorostegi-Anduaga et al.  2018 | Spain | Randomized trial study | 167 individuals  (women=59, men=108) | 53.7±7.8 | Blood pressure, fasting venous blood to analyse measurements of glucose and lipid profile, age, and cigarette smoking status. All medicines being taken were ascertained from the  participant’s physician. |
| Hohenester et al.  2018 | Germany | Observational  study | 152 individuals  (women=108, men= 44) | 44.6 ± 11.5 | Weight, height, blood pressure, heart rate, and waist circumference were determined, and routine serum chemistry was performed for sodium, potassium, creatinine, uric acid, AST, ALT, GGT, alkaline phosphatase, triglycerides, total cholesterol, LDL-c, HDL-c, HbA1c together with a complete blood count. In addition, abdominal ultrasound and bioelectrical impedance analysis. For 43 patients, additional serum parameters (albumin, adiponectin, M30, IL-8, MIP-1,  leptin, ghrelin, and TNFα) were determined. Fatty acid quantification. |
| Ikeler et al.  2018 | United  States | Randomized  Clinical trial | 92 individuals | 18 - 75 | Anthropometric measurements (height, weight, BMI, and hip and abdominal waist circumference), vital signs (blood pressure and pulse), medication review, standard of care dietary counselling, body composition measurements with dual energy x-ray absorptiometry and bioelectrical impedance analysis, resting energy expenditure, and VO2 peak fitness. Nutritional, metabolic, oxidative stress, and inflammatory  biomarkers were measured from blood and urine samples. Dietary recalls and questionnaires. |
| Lowry et al.  2018 | Canada | Prospective and longitudinal | 159 patients  (women = 82, men = 77) | 60.7±0.73 | DNA extraction and genotyping, SNP selection (ATP2B1, ACE, GLUT2, TCF7L2, ADIPOQ, FADS1, CETP, APOC3, APOA5, APOA1, MC4R, FTO).  Standard blood clinical measurements (lipids, glucose), maximal oxygen consumption (VO2max), and the continuous Metabolic Syndrome score (cMetS) (calculated by combining the weighted effects of waist circumference, triglycerides, blood glucose, and systolic blood pressure). |
| Pataky et al.  2018 | Switzerland | Prospective  study | 114 patients  (women=95, men=19) | Women: age  45.6 ± 10.5  Men: age  45.8 ± 9.9 | Body weight, % body fat, and fat-free mass, fasting glucose, HbA1c, lipids, liver parameters, and blood pressure, psychosocial well-being, and QoL of every participant. Plasma levels of N- arachidonoylethanolamide (AEA), 2-arachidonoylglycerol (2-AG), N-oleoylethanolamide (OEA), plasma N-palmitoylethanolamide (PEA), Basal metabolic rate, 3-day paper food diary, Physical activity measured by a small single-axis accelerometer, Beck Depression Inventory, Beck Depression Inventory, Eating Disorder Examination Questionnaire (EDE-Q), Impact of Weight on QoL (IWQOL-Lite ©. |
| Simonson et al.  2018 | United  States | Randomized parallel-group clinical trial | 38 patients  (women=23, men=15) | 21 - 65 | Weight, height, waist circumference, seated blood pressure, and medication doses. Clinical laboratory tests included HbA1c, fasting plasma glucose, total cholesterol, LDL-c, HDL-c, triglycerides, microalbuminuria, renal function, liver function, and haematology. Body composition was assessed by bioelectrical impedance. A 6-minute walk test was performed. The UK Prospective Diabetes Study (UKPDS) Risk Engine was used to calculate the risk of fatal and nonfatal cardiovascular events and stroke, the 36-Item Short Form Health Survey (SF-36), and the Impact of Weight on Quality of Life (IWQOL). |
| van Dammen et al.  2018 | Netherlands | Randomized controlled trial | 577 women with infertility | 18 - 39 | Weight, BMI, waist and hip circumference and ratio, systolic and diastolic blood pressure, fasting serum concentrations of glucose and insulin, and physical and mental QoL (quality of life)., HOMA-IR, triglycerides, total cholesterol, LDL-c, HDL-c, and hs-CRP, 36-Item Short Form Health Survey (SF-36), Short Questionnaire to Assess  Health-enhancing physical activity (SQUASH). |
| Watanabe et al. 2018 | Italy | Prospective randomized controlled pilot study | 22 female patients | 18 - 65 | Body weight and height, waist circumference, systolic and diastolic blood pressure, heart rate, and body composition was measured by dual x-ray absorptiometry. |
| Ballin et al.  2019 | Sweden | Randomized controlled trial | 90 old men | 70.7 ± 0.2 | 36-Item Short Form Health Survey (SF-36); resting heart rate (HR), systolic blood pressure, and diastolic blood pressure; Total cholesterol, HDL-c, LDL-c, and triglycerides. |
| Deibert et al.  2019 | Germany | Randomized single-center study | 22 individuals  (women=9, men=13) | Replacement group= 57  Lifestyle group= 54 | Sonographic findings of fatty liver, ALT; MRI and MRS measurements  performed with a 3T whole-body MRI system; abdominal fat-water imaging; liver fat content and composition measured by MRS using a single voxel PRESS (Point RESolved Spectroscopy); reconstruction of fat and water images performed using the graph cuts algorithm; segmentation of subcutaneous and internal adipose tissue, including visceral adipose tissue, muscular fat, and bone marrow conducted in the abdominal region with an active contour algorithm; liver spectra; lipid peak quantification; total fat signal ; lipid chain length, saturated lipid component, total unsaturated lipid component and fraction of unsaturated lipids; body composition (plethysmography); body weight, waist and abdominal circumference), self-reported medical history, blood pressure, glucose, and serum lipids; adipokines (i.e. leptin, adiponectin, resistin, vaspin, and fetuin A). |
| Aakre et al.  2020 | Norway | Non-randomized clinical trial | 131 individuals  (women=86, men=45) | Maximum age: 55 years | A complete biomarker set [baseline and follow-up cardiac troponins, NT- proBNP, and hs-CRP; serum glucose, creatinine, and blood lipids, HbA1c, and insulin; arterial hypertension; analyses of body composition were performed using bioelectrical impedance analysis (Inbody 720, Body Composition Analyzer, Biospace, Seoul, South Korea); blood pressure; HOMA-IR; carotid femoral pulse-wave velocity (cfPWV) by The SphygmoCorVR system (AtCor Medical, Sidney, Australia) and a single high-fidelity applanation tonometer (MillarVR), and waist-to-hip ratio. |
| Calvo-Malvar et al.  2021 | Spain | Randomized, controlled, parallel-arm, community-focused dietary intervention trial | 250 families  (720 individuals) | 18 - 85 | Weight, body mass index, lipid profile (total cholesterol, and LDL-c; C- reactive protein [CRP]; [TNF-α]), glucose and insulin resistance levels (fasting plasma glucose and HOMA-IR, systolic and diastolic blood pressure; sociodemographic characteristics; tobacco and alcohol consumption; medication; anthropometric data; blood pressure; health- related quality of life; adherence to the Atlantic diet; a 3-day food record including two weekdays and either a Saturday or Sunday. |
| Haufe et al.  2021 | Germany | Prospective, randomized, and single-blind (assessor blind) trial | 314 individuals  (women=178, men=136) | Intervention group  48.3±67.9  Control group  47.8± 8.5 | Electrocardiogram, case history, and physical examination, body weight, waist circumference, height; blood pressure, fat mass, and exercise capacity; transaminase measurements and calculation of the aspartate aminotransferase-to-platelet ratio index (APRI) score and the Fibrosis-4 score; questionnaires on anxiety and depression severity; health-related quality of life (short-form 36); daily physical activity (Freiburger Physical  Activity Questionnaire and work ability index). |
| Soldevila-Domenech et al.2021 | Spain | Longitudinal analysis of PREDIMED-Plus  (multi-centre randomized parallel-group) | 487  (women = 244, men 243) | Men 55 - 75  Women 60 - 75 | Cognitive performance: Short-term and long-term auditory memory, using the Rey's Auditory-Verbal Learning Test (RAVLT), Visuoconstructive praxis, short- and long-term visuospatial memory  and visual perception; Symbol Digit Modalities Test (SDMT); Premorbid intelligence quotient; Folstein Mini-Mental State Examination (MMSE); weight, height, hip and waist circumference; BMI; blood pressure; blood glucose, HbA1c, and lipid levels: triglycerides, total cholesterol, HDL-c, and LDL-c. Adherence to the er-MedDiet was evaluated with a 17-item er-MEDAS questionnaire; Beck’s Depression Inventory-II. |

RYGB: Roux-en-y gastric bypass; IMWM: Intensive medical weight management; BMI: Body mass index; HDL-c: High density lipoprotein cholesterol; LDL-c: low density lipoprotein cholesterol; HOMA-IR: Homeostasis Model Assessment of Insulin Resistance; VO2: volume of oxygen; hs-CRP: High sensitive C-reactive protein; HbA1c: Glycosylated hemoglobin A1c; AST: Aspartate transaminase, ALT: Alanine transaminase, GGT: gamma-glutamyl transferase; IL8: Interleukin-8; MIP-1: Macrophage inflammatory protein; TNF-α: Tumor necrosis factor alpha; IL-1: Interleukin-1; IL-6: Interleukin-6; PAI-1: Plasminogen activator inhibitor-1; ICAM-1: Intercellular adhesion molecule 1; VCAM-1: Vascular cell adhesion molecule 1; QUICKI: Quantitative Insulin Sensitivity Check Index; MetS: Metabolic Syndrome; MRI: Magnetic resonance imaging; MRS: Magnetic resonance spectroscopy.

**Table S2A.** Multidisciplinary therapies reported in the selected studies

| **Authors** | **Multidisciplinary therapy** |
| --- | --- |
| Esposito et al.  2003 | Control group received general oral and written information about healthy food choices and exercise at baseline and monthly visits, without specific individualized programs. Women in the intervention group received specific orientation of reduction in weight of 10% or more. The program included education on caloric restriction, personal goal setting, and self-monitoring (food diaries) through a series of monthly small-group sessions. Behavioural and psychological counselling was also offered. |
| Womble et al., 2004 | Women were randomized into two groups aimed at weight loss. In the eDiets group, participants were followed by a multidisciplinary team through an Internet-based weight loss program that included nutritionist counseling, personalized dietary plans, social support, psychological meetings, and physical activity recommendations tailored to the participants’ fitness levels. In the other group, the intervention was based on the book *LEARN Program for Weight Management 2000.* These participants were encouraged to follow a pre-established diet plan and received general guidance to increase physical activity. Similarly, they attended group sessions with a psychologist. |
| O’Brien et al.  2006 | The program involved guidance on proper eating habits and physical activity. Patients’ adherence to prescribed tasks, such as maintaining a food diary, and their overall compliance with scheduled appointments were regularly evaluated. Participants were randomly assigned to one of three groups: conventional care, an intensive nonsurgical program, or laparoscopic adjustable gastric banding. Nonsurgical Program: This approach focused on behavioral modification, a very-low-calorie diet, and pharmacotherapy, combined with education and professional support to promote appropriate eating and exercise behaviours. Surgical Program: This involved the laparoscopic adjustable gastric banding procedure (LAP-BAND System). |
| Tuthill et al.  2007 | Intervention group: dietary advice + exercise advice. |
| Hofso et al.  2010 | Surgery group or intensive lifestyle intervention at a rehabilitation centre. Both groups included dietician. The intensive lifestyle group was allocated to rehabilitation centre. |
| Kalter-Leibovici et al.  2010 | The lifestyle interventions incorporated a team-based approach involving dietitians who provided both individual and group nutritional counselling. Physical activity specialists led group exercise sessions in the intensive intervention. Cultural considerations were integrated into the program design to enhance relevance and engagement. Additionally, educational materials in Arabic were supplied to support learning and behaviour change. This multidisciplinary effort combined nutritional guidance, physical activity support, and culturally tailored education to promote healthier lifestyles. |
| Thomson et al.  2010 | Randomized to one of three 20-week lifestyle programs: diet only, diet and aerobic exercise, or diet and combined aerobic-resistance exercise. |
| Straznicky et al.  2011 | Randomization to 12-wk hypocaloric diet alone (n =8) or together with aerobic exercise training (n=10), followed by a 4-month weight maintenance period. |
| Bischoff et al.  2012 | The subjects underwent a multidisciplinary non-surgical weight loss program (OPTIFAST52- OF52 program) consisting of five phases lasting 52 weeks. They were monitored by psychologists, physician, nutritionists, and physiotherapists. |
| Vetter et al.  2013 | In the Brief Lifestyle Counseling group, care involved both primary care providers (PCPs) and auxiliary healthcare personnel trained as lifestyle coaches (typically medical assistants). The Enhanced Brief Lifestyle Counselling group received a more comprehensive intervention, which included pharmacological therapy (sibutramine or orlistat) or meal replacements, alongside the same behavioral counseling and PCP visits provided to the other groups. |
| Waters et al.  2013 | - Diet-behavioural therapy for all participants. Participants randomized to the diet-exercise group also underwent supervised aerobic/resistance exercise. After the 1-year lifestyle intervention, participants remained in the community with no contact by study personnel, until the 30-month follow-up period. |
| Donini et al.  2014 | Groups were: Standard nutritional treatment (SNT) or an integrated and multidisciplinary obesity treatment program (NPPRP). Nutritional assessment, psychological and physical evaluation were applied in both groups. SNT received a personalized diet, general physical activities guidelines and no psychological intervention. NPPRP received personalized diet, physical reconditioning, cognitive behavioural psychotherapy and educational activities. |
| Halperin et al.  2014 | Subjects were allocated either to RYGB or to Why WAIT. Why WAIT was a 12-week multidisciplinary program for weight control and intensive diabetes management. Why WAIT’s multidisciplinary approach included an endocrinologist, dietician, exercise physiologist, mental health provider, and diabetes nurse educator. |
| Nestvold et al.  2014 | This approach involved collaboration between nutritionists, specialized nursing staff, and educators, emphasizing a coordinated effort to support behavioral change and prepare patients both physically and psychologically for bariatric surgery. |
| Courteix et al.  2015 | Participants were randomized into three groups with different intensities of exercise (intense resistance, intense endurance, moderate mixed). They underwent supervised exercise for three weeks, followed by unsupervised physical activity for one year. All were given a restrictive diet. |
| Ding et al.  2015 | Participants were randomized to either laparoscopic adjustable gastric banding (LABG) or to an intensive medical weight management and diabetes program (IMWM), which involved a multidisciplinary team with a nutritionist, an exercise physiologist, an endocrinologist, a psychologist, and a diabetes nurse educator. |
| Jamar et al.  2015 | The interdisciplinary weight loss program combines exercise training (aerobic plus resistance training) with clinical, nutritional and psychological therapy. All measurements were performed before and after intervention. |
| Serrano-ferrer et al.  2016 | Dietary management and physical activity. Individuals with Metabolic Syndrome were randomly assigned to different exercise groups, which included either high-intensity resistance training, high-intensity aerobic training, or moderate-intensity training combining both exercise types. |
| Jahangiry et al.  2017 | The intervention in this study occurred via the website/app and e-mails. Both groups received information about metabolic syndrome and cardiovascular risk, but the intervention group was logged in to the “My Healthy Heart Profile” app, where they maintained contact with researchers, had access to information, and had assessments of their measurements. They also received a dietary intervention. The control group (waiting list) received general healthy lifestyle information every three weeks by email. |
| Panosian et al.  2017 | Surgery group: RYGB or multidisciplinary IMWM program for patients with obesity and type 2 diabetes: WhyWAIT (Weight Achievement and Intensive Treatment), which included dietary and behavioural approach, diabetes educators and exercise specialists. |
| Budui et al.  2018 | The participants were admitted to a multidimensional rehabilitation program (MRP) with multidisciplinary follow-up and group and individualized interventions. The program focused on nutrition, psychology and physical capacity. |
| Castro et al.  2018 | A nutritional intervention based on a commercial weight-loss program (PNK method®) + encouragement to exercise on a regular basis using a formal exercise program. |
| Freitas et al.  2018 | -Adults with grade II obesity and asthma were randomly assigned to one of two intervention groups: a weight loss program + exercise program (WL + E group) or a weight loss program + sham (WL + S group).  - The WL + E group engaged in a weight loss protocol including aerobic and resistance muscle training, nutritional counselling and psychological therapies, whereas the WL + S group performed breathing and stretching exercises. |
| Gorostegi‐Anduaga et al.  2018 | There were four intervention groups, all of which received guidance on the Dietary Approaches to Stop Hypertension (DASH) diet and had meetings with a nutritionist every two weeks. The groups differed based on the type of exercise intervention. Each group had different intensities and durations, and three of the four groups had supervised sessions with specialists. |
| Hohenester et al.  2018 | The patients were followed in the lifestyle intervention program by nutritionists, physical education instructors, physician and psychologists for 52 weeks. The intervention took place with weekly group and individual counseling sessions. Behavioral therapy was aimed at implementing a controlled diet without weight gain. The individuals were instructed to consume a meal replacement formula for twelve weeks, and then the food was reintroduced and controlled for weight loss. |
| Ikizler et al.  2018 | Participants were randomized to four groups consisting of either calorie restriction associated with aerobic exercise, calorie restriction alone, aerobic exercise alone or usual care. The dietary intervention was based on the implementation of a diet with a daily energy restriction of 300-500 kcal. The physical intervention was based on practicing physical activity for 35-40 minutes three times a week. |
| Lowry et al.  2018 | A personalized lifestyle intervention, guided by a nutritionist, primary care physician, and kinesiologist, lasting one year. An individualized diet based on the principles of the Mediterranean diet was proposed, together with a combination of aerobic, flexibility, and resistance exercises, to achieve sustainable behavioral changes. |
| Pataky et al.  2018 | The 12-month multidisciplinary intervention involved a physician, nurse, psychologist, and nutritionist. The initial phase included a four-day consecutive educational program, followed by outpatient follow-up and educational and motivational group sessions every 2-3 months. The individuals were also followed up monthly by a health care provider (HCP) to adjust their physical activity and diet and resolve any difficulties in following the plan. |
| Simonson et al.  2018 | The study included two groups: Roux-en-Y gastric bypass (RYGB) surgery and intensive medical diabetes and weight management (IMWM). The multidisciplinary approach included an endocrinologist, registered dietitian (applying hypocaloric diet), exercise physiologist (guiding 300 min/week of individualized exercise), mental health provider, and diabetes nurse educator (certified). |
| van Dammen et al.  2018 | Women with infertility were randomized to either the lifestyle intervention group or the classic infertility treatment group, following Dutch guidelines. Participants in the lifestyle intervention group received both dietary and physical activity interventions. They were accompanied by nutritionists and nurses trained in motivational counselling techniques. |
| Watanabe et al.  2018 | Individuals were allocated to two groups, which differed only in their mangosteen supplementation (400 mg/day). Both groups were instructed to follow a low-calorie diet and practice moderate-intensity physical activity. Participants had monthly meetings with a nutritionist. |
| Ballin et al.  2019 | Participants in the control group were instructed to maintain their lifestyle habits. Individuals randomized to the intervention group underwent 10 weeks of progressive vigorous interval training. All individuals received personalized lifestyle guidance focusing on physical activity and diet 12 months before the study. |
| Deibert et al.  2019 | Two groups: meal replacement group (MR-G) and lifestyle change group (LC-G). Physical exercise was performed as a group session once a week during the first six weeks and twice a week thereafter. The group-based exercise sessions were led by a sport physiologist. |
| Aakre et al.  2020 | Participants were assigned either to the intensive lifestyle intervention (ILI) group, based on dietary intervention and physical activity, or the group assigned to Roux-en-Y gastric bypass, preceded by 7 weeks on a low-calorie diet. |
| Calvo-Malvar et al.  2021 | The family randomized for the control group was instructed to maintain their usual routine and habit. The families randomized to the intervention group were followed up for six months by a nutritionist to incorporate the Atlantic diet into their daily lives. The intervention included educational sessions, recipe books, cooking classes, and a supply of foods typical of the diet. |
| Haufe et al.  2021 | The subjects were randomized to either the waiting-list control group or the intervention group. The intervention group consisted of supervised exercise for six months, with nutritional guidance. |
| Soldevila-Domenech et al.  2021 | The participants were randomized to two groups: the intervention group (IG) and the control group (CG). The IG consisted of a lifestyle intervention based on the Mediterranean diet with a daily calorie reduction of 30% (er-MedDiet), behavioral support for weight loss goals, and the promotion of physical activity. The CG, on the other hand, was guided by traditional Mediterranean diet recommendations without energy restriction. Both groups were given extra virgin olive oil (1L/month) and occasional almonds (125g/month) to increase adherence to the study. |

Dietary Approaches to Stop Hypertension (DASH); IMWM: Intensive Medical Diabetes and Weight Management; LAGB: Laparoscopic Adjustable Gastric Band;
MedDiet: Mediterranean Diet; PCP: Primary Care Provider; RYGB: Roux-en-Y Gastric Bypass; Why WAIT: Weight Achievement and Intensive Treatment.

**Table S2B.** Description of different approaches of multidisciplinary interventions.

| **Authors** | **Sessions**  **(frequency and duration)** | **Length**  **(short-long term)** | **Focus (individual/ group)** | **Follow-up** | **Drop out** |
| --- | --- | --- | --- | --- | --- |
| Esposito et al.  2003 | At baseline and during monthly visits, all participants received general oral and written information about healthy eating and physical activity, but no individualized plans were provided to the control group. Women in the intervention group received: specific weight-loss goals (≥10% body weight reduction), education on calorie reduction, behavioral strategies such as goal setting and self-monitoring (food diaries). They had monthly sessions with the nutritionist and exercise trainer on first year and bimonthly sessions for the second year. Every month small-group sessions were done, and behavioral and psychological counseling was also applied. | 2 years | Individual and group | No | 4,16 %  (n=5) |
| Womble et al., 2004 | *eDiet* group: women had individual virtual consultations with a nutritionist and received a personalized diet with energy values based on their BMI. They also received customized grocery lists. Participants received physical activity guidelines tailored to their self-reported cardiovascular endurance and muscle strength levels. Social support was provided through online meetings moderated by a professional, support groups in online forums, a 24-hour help desk staffed, and email reminders about the program and its goals, as well as newsletters about diet and fitness. The women had a 20-minute meeting with a psychologist at the beginning of the study and then at four different times during the study.  Weight Loss Manual (*LEARN program*): women were instructed to follow a pre-established diet of 1200 to 1500 kcal/day, increase physical activity (walking up to 30 minutes/day), and practice other behaviors for weight control. After 16 weeks, they received the *Weight Maintenance Survival Guide*. Women in this group had meetings with the psychologist on the same schedule as the *eDiet* group. | 1 year | Individual | No | 34% (n=16) |
| O’Brien et al.  2006 | Nonsurgical Program: Patients were seen by a physician every two weeks throughout the very-low-calorie diet phase, and then every four to six weeks during the remainder of the study. No patient had intervals longer than six weeks between visits.  Surgical Program: Surgery was conducted within one month after randomization. The surgeon monitored patient progress every four to six weeks and adjusted the saline volume in the gastric band during office visits based on standard clinical guidelines.  Common Program: All participants received guidance and encouragement to adopt healthy lifestyle habits, including proper dietary practices and increased physical activity. They were advised to aim for at least 200 minutes of exercise per week | 2 years | Individual | No | 10%  (n=8) |
| Tuthill et al.  2007 | Participants in the intervention group attended monthly evening group sessions, where they received nutritional guidance from a dietitian and physical activity recommendations from a physiotherapist. Additionally, they were provided with Polar heart rate monitors (to track heart rate during exercise) and were given personalized exercise plans. | 6 months | Individual and group | No | 17,64%  (n=12) |
| Hofso et al.  2010 | -Surgical group: Patients concluded a low-calorie diet in 3–6 weeks before surgery. During follow-up, a bariatric surgeon examined patients at 6 weeks postoperatively, while a dietician saw quarterly.  - The 1-year lifestyle program included 4 visits to rehabilitation, each lasting between 1 - 4 weeks. During the visits, the daily schedule combined 3–4 h of structured physical activity with psychosocial intervention. Between visits, patients received biweekly phone calls for support. They were also encouraged to track their eating habits and physical activity independently, and to see their general practitioner every four weeks for consultation and weight monitoring. | 1 year | Individual and group | No | 4,54%  (n=3) |
| Kalter-Leibovici et al.  2010 | -The intensive lifestyle intervention participated in a monthly individual counseling session and a monthly group meeting (with dietitian). Also, they participated in monthly group sessions (twice) with a physical activity instructor. They were advised to maintain personal logs of daily physical activity and 24-hour records of dietary intake in the 3 days before personal sessions with the dietitian. Fifteen women were scheduled per dietary or physical activity group session. During the Ramadan, no meetings were schedule. - Women assigned to the moderate lifestyle intervention (control group) had three individual counseling sessions with a dietitian: at baseline, after 6 and 12 months. They also had 2 educational group sessions with a dietitian in the first month of the intervention. - The duration of both interventions were 1 year. The intervention targets were attainment of at least a 7% reduction of initial body weight and at least 150 minutes per week of physical activity (moderate intensity). | 1 year | Individual and group | No | 13,43%  (n=27) |
| Thomson et al.  2010 | (1) a diet-only group (DO), which followed an energy-restricted, high-protein meal plan totaling 6,000 kJ/day; (2) a diet plus aerobic exercise group (DA), which combined the dietary plan with five weekly walking or jogging sessions; and (3) a diet plus combined exercise group (DC), which included the same diet along with a mixed exercise routine consisting of three walking/jogging sessions and two resistance training sessions per week. | 20 weeks | individual | No | 47,87%  (n=45) |
| Straznicky et al.  2011 | Dietary weight loss alone (WL) or dietary weight loss combined with aerobic exercise (WLEX). The exercise protocol consisted of cycling sessions performed every other day, lasting 40 minutes each, at an intensity corresponding to 65% of the individual’s maximum heart rate. Following this phase, participants entered a 4-month weight maintenance period, during which caloric intake was gradually adjusted to maintain energy balance. They were advised to continue following the Dietary Approaches to Stop Hypertension (DASH) eating pattern and received general guidance on physical activity. Monitoring was conducted through follow-up visits every three weeks. | 12 weeks + 4month | individual | No | Not reported |
| Bischoff et al.  2012 | A group of 8 to 15 people met weekly for approximately three and a half hours throughout the program. The intervention occurred in five phases. The first phase consisted of one week of follow-up to check the inclusion and exclusion criteria, followed by the second phase of 12 weeks, with the replacement of meals with a formula totaling 800kcal/day. The third phase lasted six weeks and aimed to gradually replace the formula with solid food again, without altering the energy value. The fourth phase was an individual energy adjustment aimed at stabilizing weight and lasted seven weeks. The fifth and final phase consisted of 26 weeks of intensive nutritional education and behavioral modification aimed at enabling individuals to achieve long-term weight control. All phases were followed up with medical examinations, exercise, behavioral therapy classes, and nutritional counseling, the amount of which varied throughout the treatment. | 52 weeks | Individual and group | No | 42%  (n= 3446) |
| Vetter et al.  2013 | -(1) Usual Care, consisting of quarterly appointments with their primary care provider (PCP), during which they received brief (5-7 minutes) guidance on weight management.  (2) Brief Lifestyle Counseling (Brief LC), which included the same quarterly PCP visit plus 10–15 minutes of monthly behavioral counseling delivered by an auxiliary health-care provider (typically a medical assistant) who was trained as a lifestyle coach.  -(3) Enhanced Brief Lifestyle Counseling (Enhanced Brief LC), which incorporated either pharmacologic treatment (sibutramine or orlistat) or meal replacements, in addition to the behavioral counseling and PCP visits, to promote greater weight loss  While all participants were given the same dietary and physical activity targets, the level of behavioral support varied across groups. They were advised to progressively increase physical activity, aiming to reach 180 minutes per week. | 2 years | Individual | No |  |
| Waters et al.  2013 | All participants received dietary and behavioral counseling aimed at achieving approximately 10% weight loss within the first six months, followed by weight maintenance for another six months. Participants randomized to the diet-exercise group also underwent supervised aerobic/resistance exercise three times a week during the intervention. | 12 months | Individual | 18 months | 38%  (n=10) |
| Donini et al.  2014 | Patients enrolled in the multidisciplinary obesity treatment (NPPRP group) were required to dedicate approximately 4 h for day (twice a week) to activities such as physical reconditioning and psychotherapy. The duration of treatment was not predetermined. Initially, a weight loss goal of 10 % was proposed.  In both groups, all patients were required to: – Nutritional evaluation once a month  – Undergo fitness and psychological status assessment each time a 10% of weight loss was reached. | 4 years | Group | No | 65,3%  (n=303) |
| Halperin et al.  2014 | *Why WAIT program*: The 12-week initiation phase included diet, physical activity, behavioral, and educational support, followed by monthly support sessions for a total of one year (maintenance phase).Two-hour weekly group were applied during the initiation phase. Participants were enrolled in small cohorts of 10-15 participants to encourage group interaction, cohesion, and support.  Patients received individualized medication and participated in supervised exercise groups along with support and educational sessions. The key components of the Why WAIT program included: (1) weekly medication adjustments; (2) a structured, modified hypocaloric diet (1500–1800 kcal) with meal replacements for breakfast and lunch during the first 6 weeks; (3) up to 300 minutes per week of graded, balanced, and personalized exercise focusing on strength training; (4) cognitive behavioral therapy; and (5) group education. This was followed by a maintenance phase consisting of monthly one-on-one counseling for the remainder of the year. | 1 year | Group | No | 11,62%  (n=5) |
| Nestvold et al.  2014 | Lifestyle Changes (12 weeks): Patients received ongoing personalized support aimed at modifying eating behaviors and increasing physical activity, starting at baseline and continuing with biweekly phone consultations conducted by a nutritionist or specially trained nurse prior to surgery. Additionally, participation in a mandatory 2-day course was required, which focused on lifestyle modifications and the impact of bariatric surgery on daily living. Post-bariatric Surgery: Patients were evaluated only once, one year after the procedure. | No (the baseline in this study was after the 12 weeks of lifestyle changes). | Individual and group | 1 year | Not reported |
| Courteix et al.  2015 | For three weeks, the participants received standardized and personalized meals at home, prescribed by nutritionists. The diet had a daily calorie restriction of 500 kcal/day, with a macronutrient composition of 15 to 20% proteins, 30 to 35% lipids, and the rest carbohydrates. About training, the individuals were trained every day according to their allocated group:  -Re: high-Resistance-moderate-endurance, 10 repetitions performed at 70% of one maximal repetition for resistance and 30% of VO2-peak for endurance training;  - rE: moderate-resistance (30%)-high-endurance (70%);  - re: moderate-resistance (30%)-moderate-endurance (30%).  Thus, 90 minutes a day were allocated to the endurance group, and 90 minutes on four days a week to resistance, totaling an average of 15-20 hours of training per week, with adjustments to the intensity. After three weeks, the subjects had to continue the training program without supervision. The meetings with the nutritionist and physical trainer occurred in the third, sixth, and twelfth months. | 1 year | Group | No | 21%  (n=19) |
| Ding et al.  2015 | Individuals allocated to the surgical intervention received a visit from the surgical team 2 weeks after the procedure. Subsequently, visits were scheduled between 4 and 6 weeks apart to review diet and symptoms. Guidance was also given on practicing physical activity as tolerated. Those assigned to the Intensive Medical Weight Management and Diabetes Program (IMWM) were accompanied by a nutritionist, an endocrinologist, a physiologist, a psychologist, and a diabetes nurse educator who, during the first twelve weeks (initiation phase), held weekly 2-hour group sessions. Each week, the patients received medication adjustments and followed a modified low-calorie diet (1500- 1800kcal/day) with a macronutrient distribution of 40-50% carbohydrates, 30% protein, and the remainder from fats, with less than 7% from saturated fat. In addition, they were encouraged to perform 300 minutes of graded exercise per week, with an emphasis on strength training. Participants also took part in cognitive behavioral intervention and group education sessions. After the initiation phase, the maintenance phase continued until the end of the one-year intervention, with monthly individual counseling. | 1 year | Individual and group | No | 0%  (n=0) |
| Jamar et al.  2015 | Patients received medical, nutritional, psychological, and physical education support. Individuals with obesity visited the endocrinologist once each month. Medical follow-up and treatment were based on an initial patient and family history, physical examination and intervention in any health problems that had developed over the course of the therapy. Nutritional intervention was carried out once a week for 60 minutes. The focus of the nutritional intervention was autonomy in food choices and weight loss. Psychological intervention was performed weekly lasting 60 minutes, to discuss conditions associated with obesity, such as dissatisfaction with body image, depression, anxiety, and eating disorders. The combined exercise-training program was performed 3 times per week, including 30 minutes of aerobic training plus 30 minutes of resistance training per session. With the challenge of overcoming the barrier, multidisciplinary monthly interdisciplinary interventions were performed linking 2 therapies in one intervention, which were planned and implemented by professionals from different areas together. | 26 weeks | Individual and group | No | 0%  (n=0) |
| Serrano-Ferrer et al.  2016 | Participants took part in a 3-week residential program, continuing the intervention independently at home. During the residential phase, they were provided with daily personalized meals designed by dietitians to achieve a caloric deficit of 500 kcal per day. The physical activity regimen included both resistance and endurance training, performed for 90 minutes, 4 to 5 times per week. Resistance training involved 8 exercises using free weights and conventional strength equipment, completed as 3 sets of 10 repetitions each. Endurance training consisted of one weekly session combining aquagym, cycling, and walking or running, depending on the targeted intensity. The groups were divided into:  Re: high-resistance training (70% of one-repetition maximum) combined with moderate-intensity endurance activities (aerobic exercise at 30% of peak oxygen uptake);  rE: moderate-resistance training (30% of one-repetition maximum) combined with high-intensity endurance exercise (70% of peak oxygen uptake);  re: moderate resistance and moderate endurance training (both at 30% intensity). | 6 months | Individual | No | 13%  (n=13) |
| Jahangiry et al.  2017 | The intervention in this study took place online. An interactive program called “My Healthy Heart profile” was used. At the beginning of the study, the participants received an adapted calorie-restricted diet from a nutritionist. The program contained a homepage that provided basic guidance on cardiovascular risk factors and was updated twice a month. Users could also send messages with questions and receive a response within 24 hours. The program provided feedback on Framingham stroke risk, as well as anthropometric and metabolic assessments, with each record provided. The control group, those on the waiting list, received emails every 3 weeks with information on MetS, healthy eating, physical activity, and body weight loss. | 6 months | Individual and group | no | 28.12%  (n=45) |
| Panosian et al.  2017 | - Surgical intervention (gastric bypass) or lifestyle intervention  - The Why Wait program included twelve weekly group sessions (10 to 15 participants), featuring: (a) medication adjustments; (b) a structured hypocaloric diet with meals replacements for breakfast and lunch during the first six weeks, alongside planned dinner menus; (c) cognitive-behavioral therapy; (d) group educational activities; and (e) an individualized exercise regimen initially targeting up to 300 minutes per week. The exercise component combined aerobic, resistance, and flexibility training, delivered through twelve supervised 60-minute sessions per week. Exercise counseling was progressive, aiming to increase both duration and intensity of physical activity gradually on a weekly basis. Following the intensive phase, a maintenance period involved monthly individual counseling sessions throughout the first year, with continued follow-up in the second year according to clinical care guidelines. | 1 year | Individual and group | 2 years | 25,58%  (n=7) |
| Budui et al.  2018 | In the multidimensional rehabilitation program (MRP), participants underwent assessments and follow-ups with doctors, a nutritionist, a psychologist, and a physical activity professional. After an initial nutritional assessment, the individuals received a personalized diet plan with a calorie deficit of 800-1000 kcal per day and a macronutrient distribution of 50-55% carbohydrates, 20% proteins, and 25-30% fats. Daily educational meetings were organized to help individuals improve their lifestyle and restore healthy eating habits. Once a week, there were individual sessions and two group meetings with an experienced psychologist. The psychological approach was based on cognitive-behavioral therapy for obesity and the systemic/strategic approach. Regarding physical exercise, two groups were formed based on fitness level and comorbidities. The groups exercised for two hours a day, one hour in the morning and one hour in the afternoon. The sessions included 5 to 10 minutes of warm-up, 30 to 40 minutes of core work, 5 to 10 minutes of cool-down, and concluded with 5 to 10 minutes of stretching. The cycle ergometer or treadmill was used for aerobic exercise and was adjusted according to individual capacity. For strength and muscular endurance exercises, ankle and wrist weights, elastic bands, dumbbells, or the individual's body weight were utilized, with a total of 10 to 15 sets of 12 to 25 repetitions performed. Once a week, individuals were assessed for anthropometry. | 3 weeks | Group | No | No |
| Castro et al.  2018 | Participants took part in a structured weight-loss program and had schedule check-ins with the research team approximately every 15 ± 2 days. On four specific occasions, they received personalized dietary counseling, motivational support and guidance to maintain regular physical activity. A phone-based follow-up system was also established providing participants with a dedicated contact number to address questions or concerns throughout the intervention. | 4 months | Individual | No | 0%  (n=0) |
| Freitas et al.  2018 | The group that received the weight loss program combined with a sham intervention (WL + S group) underwent 12 individualized sessions of hypocaloric diet counseling provided by a nutritionist and a psychologist, incorporating behavioral strategies. In addition, they participated in sham activities—such as stretching and breathing exercises—conducted twice a week over a 3-month period.  The weight loss plus exercise group (WL + E group) followed the same dietary and behavioral intervention, with the addition of a structured exercise program. This included supervised aerobic and resistance training sessions twice per week for 3 months, along with general physical activity recommendations. All exercise sessions were overseen by a physiotherapist.  Following baseline assessments, both groups also completed an educational program consisting of four 90-minute classes held twice a week, totaling 6 hours of instruction. | 3 weeks | Individual and group | No | 7,27 %  (n=4) |
| Gorostegi‐Anduaga et al.  2018 | All participants followed the DASH diet (Dietary Approaches to Stop Hypertension) with a daily restriction of 25% of energy expenditure, aiming for weight loss of approximately 0.5 kg to 1.0 kg per week. The diet consisted of 55% carbohydrates, 15% protein, and 30% fat. The Attention Control group had the recommendation of 30 minutes of moderate-intensity aerobic exercise, five to seven days per week, accompanied by some dynamic resistance exercises. The other three intervention groups participated in supervised aerobic exercise sessions conducted by an exercise specialist, twice a week on non-consecutive days. The second group was prescribed 45 minutes of moderate-intensity exercise per session, the third group was guided to perform a moderate-to-high intensity protocol for 45 minutes, and the fourth group performed 20 minutes alternating between a moderate-to-high intensity protocol.  The participants met every two weeks to be weighed, receive nutritional advice, encouragement, and guidance. | 16 weeks | Group | No | 20,1%  (n=42)  (23,40 % of males, 13,23% of females) |
| Hohenester et al.  2018 | A medical consultation was held before the intervention to assess current dietary and physical activity habits. In the initial phase (12 weeks), a commercial formula diet of approximately 800 kcal/day was provided. After that, until the 26th week, the diet was gradually replaced by normal, balanced meals, still aimed at weight loss. From the 26th week to the 52nd week, all patients were eating normal, protein-rich meals to lose weight. Throughout this period, there were weekly group and individual sessions with nutritionists, psychologists, doctors, and physical education instructors. The sessions with the psychologists consisted of reflecting on previous eating habits and learning methods and strategies for dealing with difficult situations throughout the treatment. | 52 weeks | Group | No | 20.4%  (n=31)  (15.9% of males, 22.2% of females) 231 females) |
| Ikizler et al.  2018 | Individuals who were submitted to one of the calorie-restricted groups had a diet record collected at baseline to calculate the personalized diet with a weight loss target of 1 kilo per week. Subsequently, every two weeks, other diet records were collected to analyze adherence to the diet. For those in the low-impact aerobic exercise intervention groups, 30 to 40-minute sessions were carried out three times a week. An average expenditure of 200 to 300 kcal/mile was calculated for each meeting, and the exercises varied between the treadmill, recumbent exercise bike, elliptical trainer, and a NU-step cross-trainer. Compliance with the physical activity was monitored and documented every week. | 4 months | Group | No | 15.31%  (n=19)  after randomization |
| Lowry et al.  2018 | All participants had weekly meetings with the nutritionist and a kinesiologist in the first three months, and then monthly meetings in the last nine months of the intervention. The primary physician's follow-up took place quarterly. With the nutritionist, individualized diet plans were developed based on the Mediterranean diet and the principles of behavioral change models. The meetings with the kinesiologist were aimed at preparing individualized fitness plans to improve aerobic, resistance and flexibility training. The plans created included both supervised and unsupervised activities. | 1 year | Group (classified by genetic analysis) | No | No |
| Pataky et al.  2018 | The 12-month multidisciplinary intervention that the individuals underwent consisted of at least 9 group sessions (managed by a health care provider-HCP) and 10 individual outpatient consultations. Each group session lasted from 9 a.m. to 5 p.m. and covered various topics, such as diet, eating behavior, and physical activity. At the beginning of the program, for 4 consecutive days, the participants had individual consultations for psychological, nutritional, and medical assessment, along with group sessions according to the chosen theme. Afterwards, this training (of 4 days) continued for the next 12 months, every 2 or 3 months, with different topics. In addition, each participant was accompanied by an HCP with regular monthly meetings based on a motivational interview, and a diet or physical activity could be proposed. At the end of the 12-month intervention, new group and individual sessions were held. | 1 year | Group | No | 23.6%  (n= 27) |
| Simonson et al.  2018 | Participants were randomized either to Roux-en-Y gastric bypass (RYGB) or to the multidisciplinary WhyWait (Weight Achievement and Intensive Treatment) program. In the initial phase (12 weeks) of WhyWait, the interventions occurred through two-hour group sessions in which the individuals received adjustments to their antidiabetic medication, participated in supervised exercise and didactic sessions. The physical training consisted of graded and individualized exercises with an emphasis on strength training and accounted for up to 300 min/week. The dietary intervention consisted of a modified low-calorie diet (1500-1800 kcal) and included cognitive behavioral therapy and group education. After the initial phase was completed, the maintenance phase occurred, which consisted of monthly individual counseling for nine months. | 1 year | Group | 3 years | 11.6%  (n=5) |
| Van Dammen et al.  2018 | Women in the intervention group received dietary counseling aimed at a caloric reduction of 600 kcal/day, along with guidance to engage in moderate-intensity physical activity at least two to three times per week. A pedometer was provided with a daily goal of 10000 steps. Lifestyle intervention counseling was carried out by trained professionals with degrees in nursing or nutrition and lasted 30 minutes in person. Initially, sessions were conducted four times during the first three months, twice in the last three months, and an additional four sessions were delivered via email or telephone. All professionals were trained in motivational interviewing techniques to support behavior change. | 6 months | Group | 5–7 years | 10.9%  (n=63) |
| Watanabe et al.  2018 | All the participants received a low-calorie diet with a reduction of 300 kcal/day and were instructed to practice moderate-intensity physical activity (3-6 METS), such as walking every day for 30 minutes. Once a month, the individual met with a nutritionist to assess the prescribed diet and adherence to physical activity. | 26 weeks | Group | No | 9%  (n=2) |
| Ballin et al  2019 | The progressive vigorous interval training was performed under the supervision of two instructors with bachelor's degrees in sports medicine and lasted 10 weeks. Sessions were held three times a week in groups of 8 to 10 participants. The session was structured with a 10-minute general warm-up with full-body and dynamic stretching, followed by vigorous interval training (work-to-rest ratio 40/20 seconds), and concluded with a cool-down and static stretching for 5 minutes. The dynamic exercises involved large muscle groups, and the progression of volume was applied to all individuals simultaneously, with an increase in the number of sets. All individuals, both in the control and intervention group, had previously received guidance on diet and physical activity 12 months prior to the start of the study. | 10 weeks | Group | No | 6,49 %  (n=5) |
| Deibert et al.  2019 | Meal Replacement Group (MR-G): Subjects were advised to substitute two of their daily meals with a commercially available preparation containing soy, yogurt and honey. After the initial phase, they continued the protocol by replacing one meal per day for 18 weeks.  Lifestyle Change Group (LC-G): This group took part in six weekly educational sessions focused on healthy eating and physical activity. They had individual consultations pre-intervention and then again at week 6. All sessions were delivered by professionals specialized in nutritional guidance.  Physical activity was incorporated through group exercise sessions, held once per week during the first six weeks and twice a week afterward. These group were led by a sport physiologist. Each participant was encouraged to walk mainly at a specific heart rate reflecting 60%-75% of their estimated VO2max. | 24 weeks | Group | No | 15.3%  (n=4) |
| Aakre et al.  2020 | The individuals assigned to the intensive lifestyle intervention (ILI) group took part in training sessions three days a week for the first twelve weeks. Two sessions corresponded to supervised training, lasting 60 to 90 minutes, and one session consisted of lectures on motivation, physical activity, and nutrition. In addition, the patients were given an energy-restricted diet of 1000kcal/day based on total energy. Between weeks 13 and 52, they were instructed to practice physical activity every day for 60 to 90 minutes, with monthly monitoring. The sessions alternated between group and individual sessions every two months. The patients allocated to the Roux-en-Y gastric bypass surgery (GBS) group followed a 900 kcal/day diet for seven weeks before the surgical procedure. After surgery, they were given a standardized regimen of dietary supplements and a proton pump inhibitor. | 59 weeks | Group | No | 32,35%  (n=33) |
| Calvo-Malvar et al.  2021 | The dietary intervention to include the eating habits of the Atlantic diet took place through three nutritional education sessions at the primary health care center and was guided by nutritionists. In the first session (baseline), the nutritionists provided guidance on nutritional recommendations and guidelines and the characteristics of the Atlantic diet. They also explained the benefits of physical activity. In the second session (three months), the degree of adherence was checked, some concepts were reviewed, and the demands to achieve the goal were adjusted. In the last session (at six months), lasting two hours, the professionals again explained the benefits of healthier eating, physical activity, and the characteristics of the diet being evaluated. Every three weeks, free food baskets were delivered with local foods typical of the Atlantic diet. | 6 months | Group | No | 8,1%  (n=59) |
| Haufe et al.  2021 | Participants in the intervention group were instructed to practice 150 minutes of moderate-intensity physical activity a week for six months. They were required to wear an activity monitor throughout the study. In addition, their diet was assessed and reviewed by nutritionists who guided the consumption of macronutrients and micronutrients. | 6 months | Group | No | 12.7 %  (n =40) |
| Soldevila-Domenech et al.  2021 | Intervention group: Participants were encouraged to walk at least 45 minutes per day, six days per week, and to perform static exercises focused on strength, flexibility, and balance according to specific instructions. For resistance training, participants were encouraged to develop the strength of the major muscle groups at least two days per week, with each session of 30–40 minutes.  Participants also took part in individual motivational interviews, typically lasting 15-30 minutes. Group sessions include explanations of the recipes, menus, and other characteristics of the proposed dietary intervention and lifestyle modification, with a duration of 30–45 minutes. A total of twelve group sessions were conducted over one year for the intervention. Following the first year of follow-up, participants allocated to the intervention group are scheduled for a monthly group session (30–45 min) and an individual session (15–30 min) every 3 months. | 6 years | Group | 2 years | 22.3%  (n=109) |

IMWM: Intensive medical diabetes and weight management; MetS: Metabolic syndrome; PCP: Primary Care Provider; *Why Wait program*: Weight Achievement and Intensive Treatment program.

**Table S3.** Effect of multidisciplinary treatment among adults of different populations

| **Authors** | **Impact of multidisciplinary treatment** |
| --- | --- |
| Esposito et al.  2003 | The study demonstrates that an intervention program based on a multidisciplinary approach improved cardiovascular risk factors and could provide effective and long-term weight loss. After two years of intervention with a Mediterranean-style diet and increased physical activity, individuals showed significantly greater reductions in body weight, BMI, WHR, blood glucose, HOMA-IR, triglycerides, and HDL-c compared to the control group. Reductions were observed in serum concentrations of IL-18, IL-6, and CRP, and an increase in adiponectin. In addition, improvements in dietary habits were identified, with a higher percentage of calories from complex carbohydrates, proteins, and monounsaturated fats, along with increased levels of physical activity. |
| Womble et al., 2004 | The group with a traditional manual-based approach had greater weight loss than the eDiets group. Women in the eDiets group had 0.9% weight loss after 16 weeks of intervention and only 1.1% after one year. In contrast, women in the LEARN program had 3.6% and 4.0% weight loss after 16 and 52 weeks of treatment, respectively. Despite the difference in weight loss, no differences were observed for other parameters. In both groups, significant improvements were observed in physical functioning and vitality, fewer symptoms of depression, increased cognitive restriction, and decreased (food) disinhibition and hunger. No significant differences were identified between the groups in terms of changes in blood pressure, glucose, lipids, or lipoproteins. Analysis within the groups revealed significant changes in only two variables and in the opposite direction to that expected: LDL and HDL/total cholesterol increased but returned to baseline levels at week 52. |
| O’Brien et al.  2006 | The 24-month lifestyle intervention group and the bariatric intervention group showed improvements in quality of life, health status, and body weight, but the surgical group demonstrated statistically better results in each area. The extent of weight loss was similar between the two groups at six months. However, while the lifestyle intervention group regained weight within two years, the surgical group continued to lose weight. Thus, the total reduction in initial body weight was 21.6% in the surgical group compared to 5.5% in the lifestyle intervention group. There was also a reduction in the prevalence of Mets in the surgical group from 38% to 3% at the end of the study. |
| Tuthill et al.  2007 | This study showed that lifestyle intervention, guided by a nutritionist and a physiotherapist, improved some aspects of quality of life in patients with T2DM and obesity. It was also effective in weight loss, reducing WC and cardiovascular risk. Weight loss, regardless of the group, was associated with an improvement in the SF-36 physical function score, as well as a significant increase in the mental health score among those who exercised more at 6 months compared to baseline. The reduction in HbA1c, SBP, total cholesterol and WC, and increase in HDL-c contributed to the reduction in cardiovascular risk. |
| Hofso et al.  2010 | Individuals who had undergone gastric bypass and intensive lifestyle were evaluated. In both groups, beneficial effects were observed on glucose metabolism, lipids, blood pressure and low-grade inflammation. In the surgery group, the average weight loss was 30% and the remission rates for T2DM and hypertension were significantly higher at 70% and 49% respectively. In the lifestyle intervention group, mean weight loss was 8% with remission rates for T2DM of 33% and hypertension of 23%. Thus, the surgical intervention group had a significantly greater reduction in metabolic syndrome, electrocardiographic left ventricular hypertrophy and albuminuria. |
| Kalter-Leibovici et al.  2010 | The intensive intervention group — individuals who participated in monthly individual and group sessions with a dietitian and biweekly group sessions with a physical activity instructor—presented a reduction in WC by 5.4 cm, fasting plasma glucose by 3 mg/dL, and triglycerides by 4.5 mg/dL. In contrast, the moderate intervention group—those who received only two initial educational group sessions and three individual counseling sessions throughout the year— showed a reduction in WC 3.1 cm, and a median increase of 1 mg/dL and 5.8 mg/dL in fasting plasma glucose and triglycerides, respectively. Regarding the prevalence of Mets, a reduction of 4% was observed in the intensive intervention group, while in the moderate intervention group there was an increase of 5,2%. |
| Thomson et al.  2010 | A comparison was made between interventions involving diet alone, diet combined with aerobic exercise, and diet combined with both aerobic and resistance exercise in women with obesity or overweight diagnosed with polycystic ovary syndrome (PCOS). There was a reduction in body weight independent of the exercise modality, and it was possible to observe greater weight loss associated with greater reductions in the Epidemiologic Studies Depression Scale (CES-D). Improvement in the validated PCOS Questionnaire (PCOSQ) was observed in all groups, as well as improvement in the domains of emotion, body weight, and menstrual problems. A moderate diet with energy restriction improved depression scores and health-related quality of life (HRQOL), but exercise did not offer any additional benefit compared to the diet itself. |
| Straznicky et al.  2011 | The eighteen individuals who participated in the study (8 WL and 10 WL + EX) maintained their initial weight loss of 9.3 kg. In terms of physical fitness, the WL group had no change while the WL + EW group had an increase in maximum oxygen consumption of 14 ± 6%, and this was maintained for 7 months. The 9% weight loss was associated with sympathoinhibition, a therapeutic approach to MetS. |
| Bischoff et al.  2012 | A 52-week non-surgical obesity therapy program conducted in specialized centers under ambulatory conditions was highly effective in reducing body weight and comorbidities in patients with grade I-III obesity. Weight loss was more pronounced in patients with an initial BMI >40-50 kg/m². Metabolic syndrome risk parameters and WC were significantly reduced, with the latter being more pronounced in male participants. Among patients diagnosed with hypertension, blood pressure decreased to a degree similar to that achieved with pharmacological treatment. Most participants were successful after one year of intervention, with two-thirds achieving weight loss greater than 15%. |
| Vetter et al.  2013 | The participants were allocated to three types of lifestyle intervention. The “Usual care” group consisted of quarterly primary care provider (PCP) visits for weight management education. The “Brief Lifestyle Counseling” group, in addition to quarterly PCP visits, had monthly behavioral counseling with a trained auxiliary health care provider. The “Enhanced Brief Lifestyle Counseling” group, in addition to the treatments described above, used weight-loss medication or meal replacements. This last group lost more weight than “Usual Care”, showed significant improvements in HDL-c and triglycerides in one or more evaluations, and in markers of insulin resistance over the two years, compared to the others. Analyzing the three groups, improvements in triglycerides, HDL-c, insulin resistance markers, and inflammation at the end of two years were associated with greater weight loss. |
| Waters et al.  2013 | All older individuals with obesity who participated in this study received diet-behavioral therapy with the intention to achieve a 10% weight loss in six months and maintain it for a further six months. Part of the participants were randomized to a group with supervised aerobic/resistance exercise three times a week for one year. After one year of intervention, they were followed up for a further 18 months without any guidance. It was possible to identify that after 30 months, 7% of the weight loss was maintained, and suggest that this is due to the reduced calorie intake. The maintenance of weight loss was clinically significant considering the findings of preservation of better physical performance test (PPT) scores and insulin sensitivity, and it was also possible to observe that lean body mass was maintained. However, total hip BMD decreased. The WC and SBP remained lower than at baseline. There were no adverse effects on liver or kidney function. This study had no control group. |
| Donini et al.  2014 | Weight loss and, consequently BMI, decreased more in the Nutritional and Psychophysical Reconditioning Program (NPPRP) group compared to the standard nutrition procedures (SNT) group. An improvement in eating behavior was observed in both groups, but from the beginning of the study, the NPPRP reported better quality eating habits. In addition, the Sick, Control, One Stone, Fat, Food questionnaire (SCOFF) score greater than two was more frequent in the SNT group. With regard to physical activity, a higher percentage of the NPPRP continued to practice it. One point to consider is that the NPPRP had fewer comorbidities.  Thus, the result of the latter highlights that multidisciplinary therapy is effective in maintaining the goals achieved after 4 years. |
| Halperin et al.  2014 | In both interventions analyzed, Roux-en-Y gastric bypass surgery (RYGB) and the WhyWAIT program, it was possible to observe improvements in self-reported physical and mental health status (Short Form-36), the impact of weight on quality of life (IWQOL), barriers to being active and problems associated with diabetes control (PAID). The positive results were comparable between the groups after one year. Glycemic and weight reduction occurred in the WhyWAIT program, but even though weight loss was maintained, dysglycemia rates were high during the study year. Thus, the highest probability of achieving the target level of HbA1c and fasting plasma glucose in individuals with obesity and T2DM was observed in the RYGB group. These individuals achieved glycemic control without the use of hypoglycemic drugs, and similar improvements were observed in blood pressure control and lipid levels. The weight loss and glycemic improvements in the RYGB group were maintained for one year of follow-up. |
| Nestvold et al.  2014 | Individuals with severe obesity underwent lifestyle changes for at least three months before bariatric surgery. The control group consisted of individuals with a BMI < 25 kg/m² without associated comorbidities, who underwent other elective surgeries. Patients with obesity had a preoperative and one-year weight loss of 14.5 kg and 48.5 kg, respectively. Thus, the reduction in BMI was 5.1 kg/m² preoperatively and 16.8 kg/m²after one year. The WC at baseline was 137 cm and reduced to 101.2 after surgery. At baseline, individuals with severe obesity presented elevated levels of components C3 and C4 when compared to the control group. These markers reduced significantly in the patients with severe obesity over time, and after 1 year of the surgery. The levels were similar to those of the control group. Similar was observed for inflammatory and coagulation markers, as well as for glucose metabolism. |
| Courteix et al.  2015 | The intensive lifestyle intervention (exercise + restrictive diet) resulted in weight loss, health improvements and body composition changes. The three different types of exercise used in the study did not have different results in terms of bone mineral content (BMC) or bone mineral density (BMD). The reduction in total body lean mass, leptin and negative energy balance negatively affected total body and BMC, as well as contributing significantly and independently to the decrease in lumbar spine BMC. In addition, the reduction in total body fat mass contributed positively to total body BMC. The increase in vitamin D contributed positively to total body BMD, BMC and lumbar spine BMD. Vitamin D and calcium intake were independent and significant predictors of femoral neck. |
| Ding et al.  2015 | The Laparoscopic Adjustable gastric band (LAGB) and the intensive medical diabetes and weight management (IMWM) program had similar 1-year benefits on cardiometabolic risk, diabetes control and patient satisfaction. These characteristics should be considered when selecting treatment for individuals with type 2 diabetes mellitus and obesity. The proportion meeting the primary glycemic endpoint was 33% and 23% in the LAGB and IMWM program, respectively. Changes in DBP, cardiovascular risk scores, lipids and fitness were similar between groups, but the reduction in SBP was greater after IMWM. Weight loss was similar at 3 months, but in LAGB it was greater at 12 months. Regarding HbA1c, the results were similar at 3 and 12 months between groups. Health status, as evaluated by the Short Form-36, Impact of weight on Quality of Life and Problem Areas in Diabetes, improved similarly in both groups. |
| Jamar et al.  2015 | Interdisciplinary therapy consisting of psychological and nutritional intervention combined with exercise training reduced anthropometric measurements and body composition variables. There was a significant reduction in the intake of total calories, carbohydrates and lipids, resulting in a slight reduction in total cholesterol and a significant reduction in LDL cholesterol. In terms of inflammatory markers, there was also a reduction in PAI-1, CRP, ICAM-1, VCAM-1 and leptin. |
| Serrano-Ferrer et al.  2016 | The lifestyle intervention included controlling diet and increasing physical activity. Independent of the physical training modalities, the lifestyle intervention significantly improved effort and left ventricular (LV) rates. It also identified beneficial effects on body composition, physical conditioning, Framingham risk score, SBP, HbA1c, fasting glucose, triglycerides and HDL-c. Regarding the inflammatory profile, there was an increase in adiponectin and a reduction in PAI-1, TNF-α and CRP in the MetS population and each group randomized to the exercise modalities. |
| Jahangiry et al.  2017 | The integration of interactive e-health programs to primary care practices offered advantages for both users and administrators of the preventive program. By providing real-time interaction possibilities for the target group. |
| Panosian et al.  2017 | This study included Roux-en-Y gastric bypass versus a multi-disciplinary, group-based medical diabetes and weight management program on physical fitness and behaviors. Comparable improvements in fitness and level of physical activity were observed two years after both interventions. The results demonstrate that after surgery, there were improvements in cardiometabolic risk estimates. Greater improvements were identified in the physical function and total scores of the IWQOL-lite, as well as in oxygen saturation and heart rate, likely associated with a greater magnitude of weight loss. |
| Budui et al.  2018 | Both older and younger patients showed a significant reduction in fat mass, fat-free mass, total body weight, BMI, NC, WC, HDL-c, improvement in HbA1c and LDL-c, and an increase in uric acid and creatinine levels. A statistically significant reduction in SBP and DBP was also observed. Only in the Young group was there a significant reduction in TG. The multidisciplinary residential context represents an ideal scenario for the initial treatment of obesity in the elderly, considering that complex clinical status and comorbidities can be a limiting factor for bariatric surgery. |
| Castro et al.  2018 | The individuals with obesity were submitted to a very low-calorie ketogenic (VLCK) diet with supportive individual counseling and encouragement to exercise regularly using a formal exercise program. This intervention induced severe weight loss concomitant with reduced cravings for alcohol and food, sleep abnormalities, improved sexual functioning, and increased physical activity. Thus, this study shows that rapid and sustained weight loss and fat mass loss induced by VLCK are associated with improvements in parameters of psychological well-being and good dietary control in individuals with obesity. |
| Freitas et al.  2018 | This study reinforces the importance of physical training in reducing asthma-related comorbid conditions in adults with obesity and asthma. These individuals who underwent a weight loss program associated with an exercise program (WL + E) had the following results: a significant increase in daily steps and the number of asthma symptoms-free, a higher proportion of participants with improvements in symptoms of depression and a lower risk of developing obstructive sleep apnea, compared to the group of individuals who underwent the weight loss program with a sham exercise treatment (WL + S). In the WL + E group, significant improvements were also observed in sleep efficiency and sleep latency. |
| Gorostegi‐Anduaga et al.  2018 | Following the association of aerobic exercise and a hypocaloric DASH diet in adults with overweight and obesity, reductions were observed in body mass, BMI, WHR, hip circumference, fat mass, and use of antihypertensive medication, along with increases in fat-free mass and improvements in SBP and total cholesterol. The improvement in CV risk factors after 16-week lifestyle change intervention reduced risk of suffering a CV event in the following 10 years. |
| Hohenester et al.  2018 | The multimodal lifestyle intervention in patients with severe obesity led to lasting weight loss, improvement in hepatic steatosis, inflammation, fibrosis and surrogate markers of overall a liver-related mortality. The data obtained suggests that in order to achieve improvements in liver physiology, higher magnitude of weight loss (>10%) may be a necessary. In addition, these data reinforce the point of view that weigh loss as a result of lifestyle intervention should be the key in the therapy of obesity-associated NAFLD. |
| Ikizler et al.  2018 | Healthy lifestyle interventions, such as non-pharmacological strategies, could improve metabolic health markers in patients with moderate to severe chronic kidney disease. The association of calorie restriction with aerobic exercise for four months resulted in significant, albeit modest, benefits in terms of fat mass, body weight, oxidative stress markers, and the inflammatory response. |
| Lowry et al.  2018 | The effectiveness of the lifestyle intervention was evidenced by an increase in the Healthy Eating Index-Canadian (HEI-C) and VO_2_max, and a reduction in the Metabolic Syndrome score (cMetS), as well as improvements in all components of MetS except fasting glucose. The study also found that the reduction in cMetS was moderated by common SNPs in two genes. These findings highlight the potential of gene-lifestyle interaction knowledge to better tailor health management strategies and set individualized goals during interventions. |
| Pataky et al.  2018 | A weight reduction was identified in 40% of the participants undergoing the structured, multidisciplinary, and educational lifestyle weight loss program for 12 months. A weight loss of 8,2% from baseline was associated with improvements in quality of life, cardiometabolic and psychological parameters, and eating disorders. It was found an interesting observation on plasma N-palmitoylethanolamid who might be a potential biomarker of metabolic improvements and also the severity of depression in patients with obesity. |
| Simonson et al.  2018 | Both intensive, multidisciplinary, and multimodal medical intervention and RYGB had improvements; however, the impact of weight on quality of life improved more after RYGB. Surgery resulted in a higher percentage of patients achieving non-diabetic glycemia, greater improvement in HbA1c and fasting plasma glucose, as well as greater weight loss over 3 years. |
| van Dammen et al.  2018 | In women with obesity and infertility, a lifestyle intervention improved cardiometabolic health and self-reported physical quality of life. |
| Watanabe et al.  2018 | The treatment and the control group followed a physical activity regimen and a standard caloric diet, but the treatment group received a mangosteen supplement (400mg). Adherence to physical activity and diet was assessed monthly by a trained nutritionist, with no significant changes over time or differences between groups. In the group that received the mangosteen supplement, a reduction in insulin concentrations was observed over 26 weeks, but no significant difference was found in WC and body composition. |
| Ballin et al.  2019 | Ten weeks of vigorous interval training were sufficient to improve mental aspects of health-related quality of life (in older individuals with central obesity), which is a critical aspect of healthy ageing. Positive effects were seen also on cardiometabolic risk markers. |
| Deibert et al.  2019 | A group was instructed to replace two daily meals with a commercially available soy-yogurt-honey preparation, and another group had lifestyle sessions about nutrition and physical exercise. Both groups showed comparable effects in terms of weight reduction, markers of hepatic inflammation and, body and liver fat content in NASH patients. In addition, there were changes in the quantity and quality of intrahepatic lipid (IHL) content concerning the proportion of saturated/unsaturated lipids and the fatty acid chain length. |
| Aakre et al.  2020 | The gastric bypass group (GBS) showed greater reductions in weight, fat mass, fat-free mass, skeletal muscle mass, BMI and WHR compared to the lifestyle intervention group. Consequently, the GBS group showed greater changes in cardiac troponin and C-reactive protein concentrations. |
| Calvo-Malvar et al.  2021 | A community-focused primary health center-led intervention, involving the Atlantic diet, induced a change in the family’s eating behavior and had the result of improving adiposity and the lipid profile. The intervention did not propose energy intake restrictions, but the individuals had a significant reduction in body weight, a relative reduction in total cholesterol and LDL-cholesterol. |
| Haufe et al.  2021 | Telemonitoring-guided lifestyle intervention, with of 6 months duration, improved markers of liver inflammation and liver fibrosis. |
| Soldevila-Domenech et al.  2021 | A long-term intensive lifestyle intervention with an energy-restricted Mediterranean diet, promotion of physical activity and behavioral support for weight loss goals (intervention group) and a more common care intervention with traditional MedDiet recommendations without energy restriction (control group) were applied. Greater improvements in memory were observed in the intervention group. In both groups, cardiovascular biomarkers improved after 1 and 3 years of follow-up. After 1 year, a reduction in body weight, WC, HC, total cholesterol, triglycerides, fasting plasma glucose, and blood pressure was identified, but after 3 years, DBP, fasting plasma glucose,HC, HbA1c and HDL-c mean changes were lower. At the baseline 236 individuals had obesity grade I, after 1 year, this number dropped to 203, and after 3 years, the total individuals with this degree of obesity was 206. Considering obesity grade II, at the baseline, 118 individuals had this classification dropping to 74 after 1 year and increasing to79 individuals after 3 years. |

BMD: Bone mineral density; BMI: Body mass index; CES-D: Studies Depression Scale; cardiac troponin I (cTnI); cardiac troponin T (cTnT); Cardiovascular (CV); Cardiovascular risk (CVR); continuous Metabolic Syndrome score (cMetS); Daily life physical activity (DLPA); DBP: Diastolic Blood Pressure; Dietary Approaches to Stop Hypertension (DASH); epicardial adipose tissue (EAT); FFA: Free fatty acid; GBS: gastric bypass group; glycosylated hemoglobin (HbA1c); HDL-c: High density lipoprotein cholesterol; Homeostasis Model Assessment of Insulin Resistance (HOMA-IR); HC: Hip circumference; ICAM-1: Intercellular Adhesion Molecule 1; IFN-γ: Interferon gamma; IL-1ra: Interleukin-1 Receptor Antagonist; IL-13: Interleukin 13; IL-18: Interleukin 18; IL-6: Interleukin 6; IWQOL: Impact Weight on Quality of Life; IMWM: intensive medical diabetes and weight management; LAGB: laparoscopic adjustable gastric band; LDL-c: Low density lipoprotein cholesterol; LV: left ventricular; MedDiet: Mediterranean Diet; MOG: morbidly obese group; NC: neck circumference; NAFLD: Nonalcoholic Fatty Liver Disease; NASH: non-alcoholic steatohepatitis; NPPRP: Nutritional and Psycho-Physical Reconditioning Program; PAI-1: Plasminogen Activator Inhibitor-1; PCOS: polycystic ovary syndrome; PCOSQ: polycystic ovary syndrome questionnaire; RYGB: Roux-en-Y Gastric Bypass; SBP: systolic blood pressure; DBP: diastolic blood pressure; OSA: sleep apnea; SCOFF: Sick, Control, One Stone, Fat, Food Questionnaire; SNT: standard nutrition procedures; TNF-α: Tumor Necrosis Factor α; UKPDS: United Kingdom Prospective Diabetes Study; VCAM-1: Vascular Cell Adhesion Molecule 1; VLCK: very low-calorie ketogenic diet; Why WAIT: Weight Achievement and Intensive Treatment; WHR: Waist-to-hip ratio; T2DM: Type 2 Diabetes Mellitus.; HRQOL: health-related quality of life.
